# Intracellular attenuation of BMP signaling via CKIP-1/Smurf1 is essential during neural crest induction

**DOI:** 10.1371/journal.pbio.2004425

**Published:** 2018-06-27

**Authors:** Michael L. Piacentino, Marianne E. Bronner

**Affiliations:** Division of Biology and Biological Engineering, California Institute of Technology, Pasadena, California, United States of America; University of Pennsylvania, United States of America

## Abstract

The neural crest is induced at the neural plate border during gastrulation by combined bone morphogenetic protein (BMP), fibroblast growth factor (FGF), and Wnt signaling. While intermediate BMP levels are critical for this induction, secreted BMP inhibitors are largely absent from the neural plate border. Here, we propose a morphogen model in which intracellular attenuation of BMP signaling sets the required intermediate levels to maintain neural crest induction. We show that the scaffold protein casein kinase interacting protein 1 (CKIP-1) and ubiquitin ligase Smad ubiquitin regulatory factor 1 (Smurf1) are coexpressed with BMP4 at the chick neural plate border. Knockdown of CKIP-1 during a critical period between gastrulation and neurulation causes neural crest loss. Consistent with specific BMP modulation, CKIP-1 loss suppresses phospho-Smads 1/5/8 (pSmad1/5/8) and BMP reporter output but has no effect on Wnt signaling; Smurf1 overexpression (OE) acts similarly. Epistasis experiments further show that CKIP-1 rescues Smurf1-mediated neural crest loss. The results support a model in which CKIP-1 suppresses Smurf1-mediated degradation of Smads, uncovering an intracellular mechanism for attenuation of BMP signaling to the intermediate levels required for maintenance of neural crest induction.

## Author summary

Induction of the neural crest, a highly migratory and multipotent type of stem cell, occurs in the embryo during gastrulation and is regulated by several signaling molecules, including bone morphogenetic proteins (BMPs). A conundrum is that the neural crest–forming territory of chick embryos has very high levels of BMP, while intermediate signaling levels are required for maintenance of neural crest induction. To achieve the requisite intermediate levels, our data show that the scaffolding molecule casein kinase interacting protein 1 (CKIP-1) acts upon the ubiquitin ligase Smad ubiquitin regulatory factor 1 (Smurf1) to mediate its autodegradation. Smurf1 in turn promotes degradation of BMP pathway components downstream of BMP receptors. Thus, CKIP-1 protects BMP signaling from Smurf1 activity. Consistent with this, we find that CKIP-1 loss causes reduced BMP signaling and loss of neural crest cells, while Smurf1 loss increases BMP signaling and neural crest numbers. Our results indicate that CKIP-1 and Smurf1 levels are essential to maintain neural crest induction by ensuring the appropriate intermediate downstream response despite high levels of BMP ligand expression.

## Introduction

The neural crest is a multipotent stem cell population that originates within the central nervous system of the developing embryo and then migrates away to diverse sites in the periphery. Following migration, neural crest cells contribute to multiple organ systems, including the peripheral nervous system, craniofacial skeleton, and pigmentation of the skin [1–3]. Induction of the neural crest occurs at the neural plate border during gastrulation by the coordinated actions of fibroblast growth factor (FGF), bone morphogenetic protein (BMP), and Wnt signaling pathways, which together are critical inputs into neural crest specification and differentiation gene regulatory networks [4,5]. Despite recent efforts to understand the precise spatiotemporal regulation of these signaling pathways during neural crest induction [6,7], our understanding of this process has been complicated due to species-specific differences in timing and regulatory mechanisms. While defined windows for FGF [8] and Wnt [9,10] activation in neural crest formation have been identified, the timing and levels of BMP signaling required for neural crest induction remain incompletely understood.

BMPs belong to the transforming growth factor beta (TGF-β) superfamily of secreted ligands and signal during important embryonic events including axial patterning and neural induction [11,12]. BMP ligands bind to transmembrane serine/threonine kinase receptors to initiate a phosphorylation cascade between receptors and the receptor-activated signal transduction molecules Smad1, Smad5, and Smad8 [13]. These phosphorylated receptor Smads form a complex with Smad4 and together enter the nucleus to regulate transcription of target genes [13]. The transcriptional result of BMP signaling displays differential responses dependent on the dose of signal, following the behavior characteristic of a morphogen [11]. Consistent with this morphogen model, high levels of BMP lead to nonneural ectoderm formation in *Xenopus* [14], whereas intermediate levels result in neural crest formation in both *Xenopus* [15,16] and zebrafish [17,18]. While the importance of BMP signaling in neural crest induction has been appreciated [12,19–21], the precise levels and timing remain unclear in chick compared with other systems.

During chick gastrulation, ectodermal expression of BMP2, BMP4, and BMP7 is highest in the neural plate border [22,23]. Despite high BMP expression, activated phospho-Smads 1/5/8 (pSmad1/5/8), the transcriptional mediators of BMP signaling, are only present at intermediate levels in the neural plate border [8]. This raises the intriguing possibility that BMP levels are attenuated to the intermediate levels proposed to be required for neural crest induction at the neural plate border, with higher levels observed in the nonneural ectoderm and contributing to epidermal cell fates. However, secreted BMP inhibitors, such as Chordin and Noggin, are not expressed in the vicinity of the neural plate border but rather in the distant Hensen’s node and notochord [22–25]. Notably, grafts of Hensen’s node into the area opaca in chick embryos induce neural plate border markers at short distances [19], suggesting that these inhibitors do not diffuse at a long enough range to play appreciable roles on endogenous neural plate border induction. While additional secreted inhibitors from the underlying mesoderm may influence the initiation of neural plate border induction [22,26], explant experiments indicate that they are not required to maintain presumptive neural crest fate [8,21,27], suggesting that cell-autonomous mechanisms may act more potently during this maintenance phase.

In searching for alternative mechanisms of modulating BMP signaling that do not depend on extracellular inhibitors, there are examples in the literature of intracellular attenuation. For example, casein kinase interacting protein 1 (CKIP-1, gene name PLEKHO1) is a membrane-associated scaffold protein [28,29] that functions in mature osteoblasts to modulate the activity of the E3 ubiquitin ligase Smad ubiquitin regulatory factor 1 (Smurf1)[30–32]. Together, CKIP-1 and Smurf1 suppress BMP signaling in bone cells by the coordinated degradation of Smads 1 and 5, and increased CKIP-1 during aging contributes to osteoporosis due to BMP suppression [30,31,33]. Smurf1 also plays regulatory roles in suppressing Smad activation during pattern formation and neural induction in *Xenopus* embryos [32,34].

We find that both CKIP-1 and Smurf1 are enriched in our chick neural crest transcriptome dataset [35]. Here, we explored the possibility that these intracellular proteins may be involved in regulation of BMP signaling at the neural plate border. Our results show that chick CKIP-1 is required for neural crest induction during gastrulation and prior to neurulation. CKIP-1 loss of function and Smurf1 overexpression (OE) each result in reduced BMP signaling output and reduced paired box 7 (Pax7) expression in the neural plate border domain. Our results suggest that CKIP-1 acts to suppress Smurf1-mediated Smad degradation by promoting Smurf1 autodegradation, uncovering a cell-autonomous, intracellular mechanism for BMP signaling attenuation at the neural plate border. Our results support a morphogen model in which intermediate levels of BMP activation, established by CKIP-1/Smurf1 double-negative attenuation of the downstream receptor Smads, are critical to maintain neural crest induction during gastrulation.

## Results

### Expression of BMP4 overlaps that of intracellular BMP inhibitors at the neural plate border

**Fig 1:**
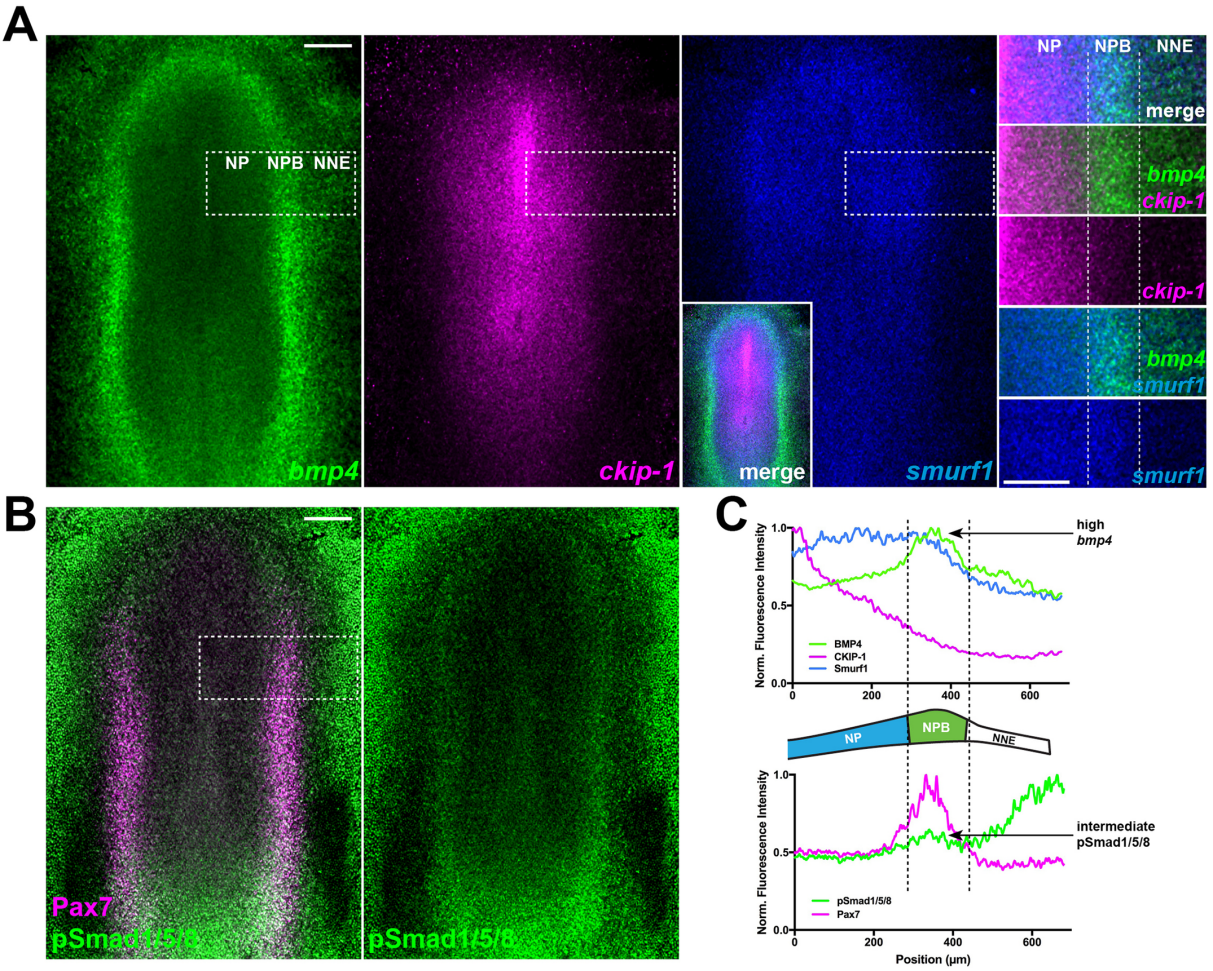
BMP4 expression overlaps with intracellular BMP inhibitor expression and intermediate Smad1/5/8 activation. (A) HCR shows expression of *bmp4*, *ckip-1*, and *smurf1* in wild-type HH6 chick embryos. (B) Immunostaining for the NPB marker Pax7 and phosphorylated Smad1/5/8 in HH6 embryos. (C) Line traces of *bmp4*, *ckip-1*, and *smurf1* expression compared with Pax7 and pSmad1/5/8 staining intensity from the boxed regions in A-B show high *bmp4* expression and intermediate pSmad1/5/8 staining in the Pax7+ NPB domain. Underlying data can be found in S1 Data. Scale bars represent 200 μm. BMP, bone morphogenetic protein; CKIP-1, casein kinase interacting protein 1; HCR, hybridization chain reaction; HH6, Hamburger-Hamilton stage 6; NNE, nonneural ectoderm; NP, neural plate; NPB, neural plate border; Pax7, paired box 7; pSmad1/5/8, phospho-Smads 1/5/8; Smurf1, Smad ubiquitin regulatory factor 1.

To determine if intracellular regulation contributes to BMP signaling during chick neural crest induction, we first examined the expression patterns for *bmp4* and two putative intracellular regulators of BMP signaling, *ckip-1* and *smurf1*, by both hybridization chain reaction (HCR) and in situ hybridization in chick embryos at Hamburger-Hamilton stage 6 (HH6; Fig 1A, S1 Fig). While *bmp4* expression was not detected in the neural plate, expression was high in the neural plate border and intermediate in the nonneural ectoderm (Fig 1A). Expression of *ckip-1* was complementary, exhibiting graded expression that was highest in the neural plate and intermediate in the neural plate border (Fig 1A). Expression of *smurf1* was detected throughout the neural plate and the neural plate border, with little expression in the nonneural ectoderm (Fig 1A). Immunostaining for activated BMP signaling transducers—phospho-Smads 1, 5, and 8 (pSmad1/5/8)—and the neural plate border specifier gene Pax7 [36,37] showed that the border displayed intermediate pSmad1/5/8 staining despite high levels of *bmp4* expression; in contrast, high pSmad1/5/8 staining was detected in the nonneural ectoderm, where *bmp4* expression is more intermediate (Fig 1B), which is consistent with previous reports [8,38]. These results were corroborated by electroporation of a BMP responsive element–driven green fluorescent protein (BRE::GFP) reporter construct [39]—which reveals intermediate activity in the neural plate border and high activity in the nonneural ectoderm (S1D Fig)—and parallel the results seen with pSmad1/5/8 staining. Together, these data suggest a scenario in which the neural plate border expresses high levels of BMP4 in conjunction with intracellular modulators of BMP signaling, which may attenuate Smad1/5/8 activation and BMP output to a level more intermediate compared to that of the nonneural ectoderm (Fig 1C). Intriguingly, we observed a small dip in pSmad1/5/8 staining levels between the Pax7-positive (Pax7+) neural plate border and the more lateral nonneural ectoderm (Fig 1B, S1D Fig). Since cranial placodes arise from the domain adjacent to the neural crest and nonneural ectoderm and require the activity of BMP antagonists [40], an intriguing possibility is that this diminished BMP activity represents the presumptive cranial placode territory.

Next, we further examined the expression of *ckip-1* as a function of time from HH4–10, ranging from neural plate border to neural crest migratory stages. The results show that *ckip-1* transcripts were detected in the neural plate and neural plate border in gastrulating embryos (HH4; S2 Fig). During neurulation, *ckip-1* transcripts were observed in the elevating neural folds (HH7), and their expression in the neural tube persisted through each stage analyzed. Transverse sections through these embryos showed that *ckip-1* transcripts are strongly enriched at premigratory stages in the neural folds (HH9), as well as in migratory cranial (HH10) and vagal (HH13) neural crest cells recognized by their human natural killer 1 (HNK-1) expression. Together, these results demonstrate that *ckip-1* is expressed in the correct spatiotemporal pattern to contribute to neural crest formation.

### CKIP-1 is necessary for neural crest formation

**Fig 2:**
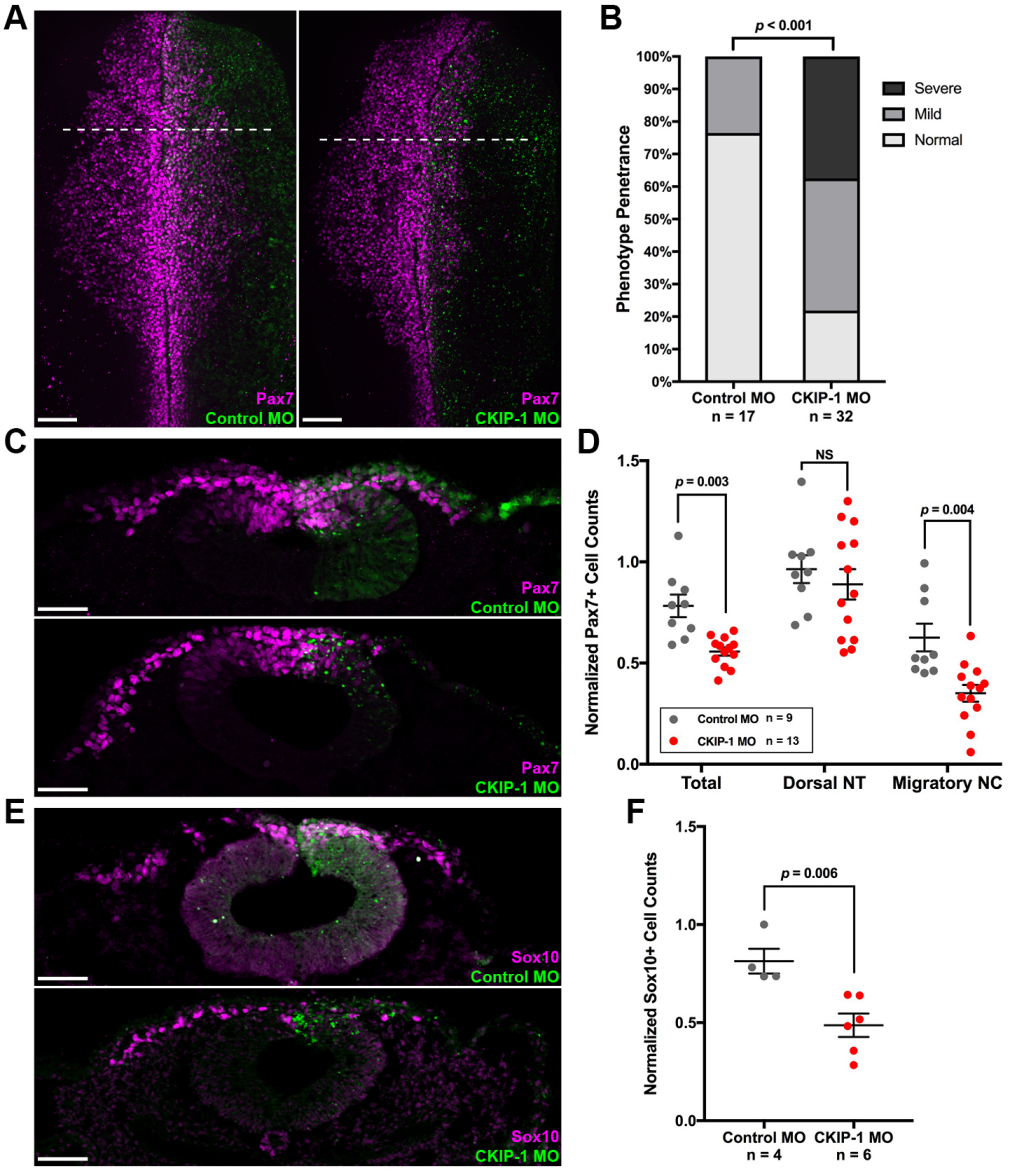
CKIP-1 is necessary for NC cell formation. (A) Unilateral HH4 electroporations were performed with FITC-conjugated random control MO or CKIP-1-targeting MO; embryos then were cultured to HH10 and immunostained for FITC and Pax7, shown in dorsal views. (B) Quantitation of phenotype penetrance for control MO—and CKIP-1 MO–electroporated embryos analyzed at HH10. Phenotypes were classified as described in S3A Fig. (C,E) Transverse sections of HH10 control MO and CKIP-1 MO embryos showing Pax7 staining (C) or Sox10 staining (E). (D,F) Quantitation of Pax7+ (D) or Sox10+ (F) cell counts following control MO and CKIP-1 electroporations, as determined from sections and normalized to counts on the uninjected side. Displayed are total cell counts, as well as the number of Pax7+ dorsal NT or migratory Pax7+ cell counts, as means ± SEMs. Underlying data can be found in S1 Data. *P* values from two-tailed Student *t* test. *n* = number of embryos analyzed per condition. Scale bars represent 100 μm (A) or 50 μm (C,E). FITC, fluorescein isothiocyanate; HH, Hamburger-Hamilton stage; MO, morpholino oligonucleotide; NC, neural crest; NS, not significant; NT, neural tube; Pax7, paired box 7; Sox10, Sry-related HMg-Box gene 10.

To determine the function of CKIP-1 during neural crest development, we performed loss-of-function analysis using a translation-blocking morpholino oligonucleotide (MO) and then analyzed expression of the neural crest marker Pax7 [36]. Unilateral electroporation of fluorescein isothiocyanate (FITC)-conjugated control MO into gastrulating chick embryos resulted in normal Pax7 expression at HH10 (Fig 2A), indicating normal induction and migration of the cranial neural crest. Conversely, FITC-conjugated CKIP-1 MO electroporations resulted in a dramatic reduction in Pax7 staining on the experimental compared to the contralateral, uninjected control side of the same embryo (Fig 2A), demonstrating a highly reproducible reduction in the number of Pax7-expressing neural crest cells (Fig 2B and S3A Fig). To test CKIP-1 MO efficiency, we next performed western blot analysis on embryos electroporated with either control MO or CKIP-1 MO (S3B Fig). The results show that CKIP-1 MO provokes a strong reduction in CKIP-1 protein levels.

To control for specificity and lack of MO toxicity, we performed rescue experiments. To this end, we subcloned the CKIP-1 open reading frame (ORF) with a C-terminal FLAG tag into a CAG promoter–driven expression vector with an internal ribosome entry site (IRES)-driven histone 2B (H2B)-red fluorescent protein (RFP) [41] and coelectroporated this construct together with CKIP-1 MO (S3C Fig). Since the translation-blocking CKIP-1 MO overlaps the start codon, 5 silent mutations were created during CKIP-1 FLAG cloning to prevent MO binding to the exogenous construct. Coelectroporation of CKIP-1 MO with CKIP-1 FLAG resulted in a partial rescue in Pax7 expression (S3D and S3E Fig), demonstrating the specificity of CKIP-1 MO and the functionality of CKIP-1 FLAG. Finally, we employed clustered regularly interspaced short palindromic repeat (CRISPR)/CRISPR-associated protein 9 (Cas9)–mediated knockout as an independent loss-of-function approach, following previously validated techniques for generating knockout effects in chicken neural crest [42,43]. Compared to a nonbinding control guide RNA (gRNA), CKIP-1 gRNA resulted in a significant depletion of neural crest markers Pax7 and Sry-related HMg-Box gene 9 (Sox9) (S3F Fig), thereby phenocopying the effects of CKIP-1 MO.

To quantitate the effect of CKIP-1 MO in the neural crest, the numbers of Pax7+ cells were counted in transverse sections through control MO—and CKIP-1 MO–electroporated embryos analyzed at HH10 (Fig 2C and 2D). The results show that total numbers of neural crest were significantly reduced in CKIP-1 morphants compared with controls, as were the numbers of migratory neural crest cells (Fig 2D). Interestingly, the number of Pax7+ cells in the dorsal neural tube were not significantly different between CKIP-1 morphants and controls. These results were corroborated using the migratory neural crest marker Sry-related HMg-Box gene 10 (Sox10) [44], which displayed a similar reduction in the neural crest cell number (Fig 2E and 2F). Importantly, overall neural tube morphology appeared normal in CKIP-1 morphants, suggesting that CKIP-1 is essential only for neural crest formation prior to HH10 but not for other Pax7+ dorsal neural tube cells.

### CKIP-1 function is required during neural plate border induction prior to HH7

**Fig 3:**
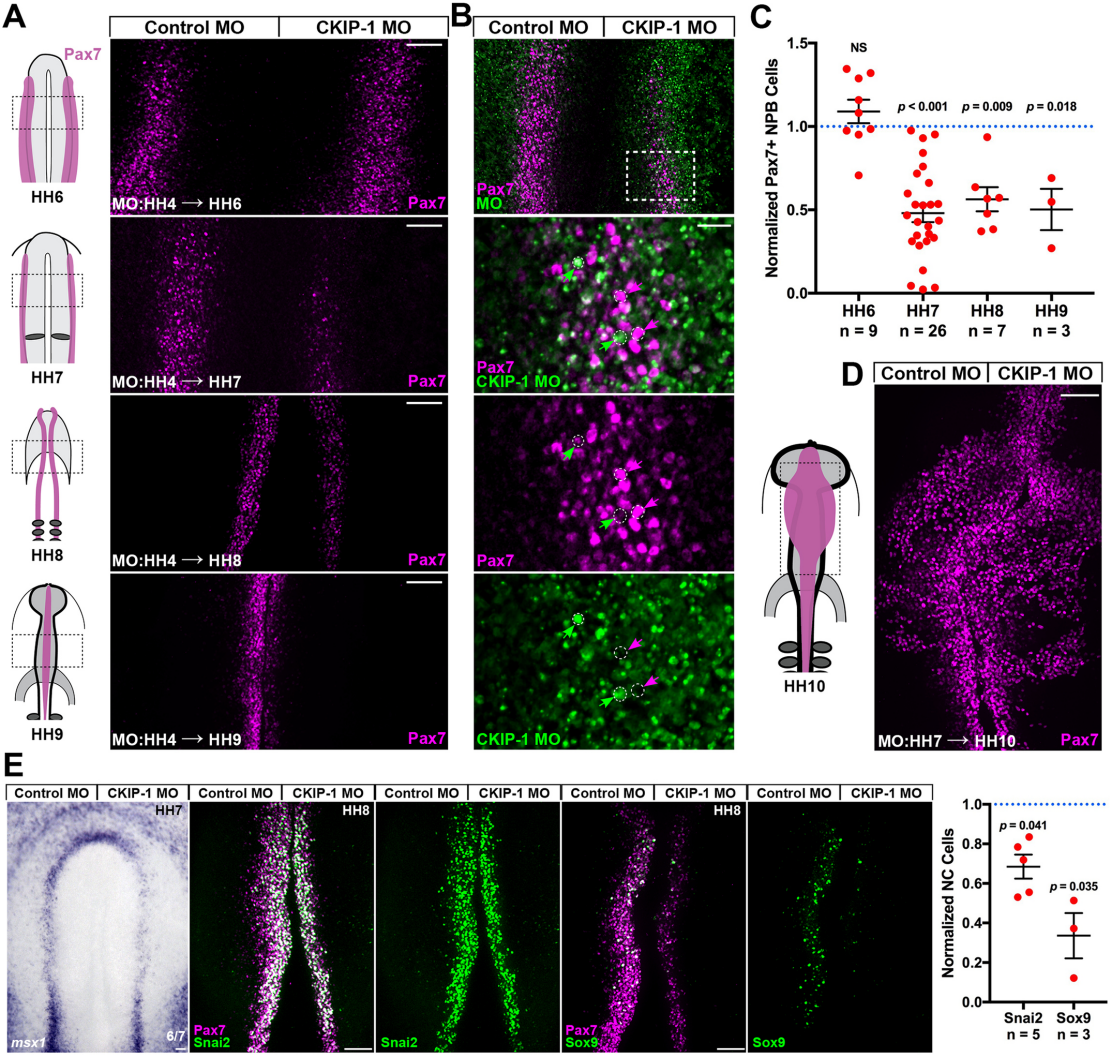
CKIP-1 is required prior to HH7 to maintain NC fate. (A) Bilateral electroporations were performed with control MO (left) and CKIP-1 MO (right) at HH4, and embryos were incubated to the indicated stages. Whole-mount dorsal views are shown following Pax7 and FITC immunostaining. (B) Single-cell examination of an HH7 embryo following electroporation described in A. Cells that received high levels of CKIP-1 MO show little Pax7 expression (green arrows), while strongly Pax7+ cells in the NPB show very little CKIP-1 MO uptake (magenta arrows). (C) Gastrulating embryos were electroporated as described in A, and normalized Pax7 cell counts are displayed with means ± SEMs at the indicated stages. *P* values from two-tailed Student *t* test. (D) Bilateral electroporations of control MO and CKIP-1 MO at HH7 results in normal Pax7 expression at HH10. (E) Bilateral electroporations of control MO and CKIP-1 MO were performed at HH4; embryos were processed for in situ hybridization for NPB marker *msx1* at HH7 or for immunostaining for NC specification markers Snai2 and Sox9 at HH8. Displayed are cell counts normalized to the control side with mean ± SEM. Underlying data can be found in S1 Data. *P* values from two-tailed Student *t* test. Scale bars represent 100 μm (A,D,E) or 25 μm (B). CKIP-1, casein kinase interacting protein 1; FITC, fluorescein isothiocyanate; HH, Hamburger-Hamilton stage; MO, morpholino oligonucleotide; Msx1, msh homeobox 1; NC, neural crest; NPB, neural plate border; NS, not significant; Pax7, paired box 7; Sox9, Sry-related HMg-Box gene 9.

The reduction in neural crest cells observed at HH10 likely reflects neural crest loss earlier in development. To determine the timepoint at which CKIP-1 functions in neural crest formation, we performed bilateral electroporations of control and CKIP-1 MOs at stage HH4 and assayed for Pax7 expression at stages HH7–HH9 (Fig 3A). Interestingly, Pax7 expression in the neural plate border was reproducibly reduced as early as HH7 (Fig 3A). In addition, Pax7 quantitation in CKIP-1 morphants revealed a significant reduction at HH7, HH8, and HH9 but not before the neural plate border is resolved at HH6 (Fig 3C). Finally, electroporation of CKIP-1 MO after HH7 resulted in normal Pax7 expression (Fig 3D).

Due to the mosaicism inherent in electroporation experiments, we performed a higher magnification examination of the neural plate border in morphant embryos. Cells that received high doses of CKIP-1 MO (Fig 3B, green arrows) showed little Pax7 expression, while cells with robust Pax7 expression showed little CKIP-1 MO uptake (Fig 3B, magenta arrows). Together, these results indicate that CKIP-1 function is required prior to HH7 for normal Pax7 expression in the neural plate border—suggesting that CKIP-1 contributes to neural plate border fate maintenance or cell survival—but not for later neural crest specification and migration. We next assayed cell proliferation and cell death in CKIP-1 morphants at HH7. CKIP-1 morphants showed normal immunostaining for phospho-histone H3 and for cleaved-caspase 3 (S4 Fig), indicating that cell proliferation and survival at the neural plate border are unaffected by loss of CKIP-1.

We confirmed these effects on the presumptive neural crest by performing in situ hybridization for neural plate border marker Msh homeobox 1 (*msx1*) and with immunostaining for neural crest specification markers Snai2 and Sox9 in CKIP-1 morphants. The results show reduced expression of each marker following CKIP-1 loss of function (Fig 3E, right side) when compared to the contralateral electroporation control (Fig 3E, left side). Neural crest specification occurs during neurulation in chick, and bona fide specification markers such as forkhead box D3 (FoxD3) and Snai2 are first detectable at HH8 [4,45]. However, we observe that CKIP-1 loss has no effect on initial Pax7 expression at HH6 but decreases expression of neural plate border markers Pax7 and, to a lesser extent, *msx1* at HH7 (Fig 3). This suggests that CKIP-1 is required during induction to maintain the neural plate border, consistent with the two-step model for neural crest induction [27,46], and acts downstream of earlier inductive signals such as FGF and Wnt [8,27].

### BMP signaling requires CKIP-1 function

**Fig 4:**
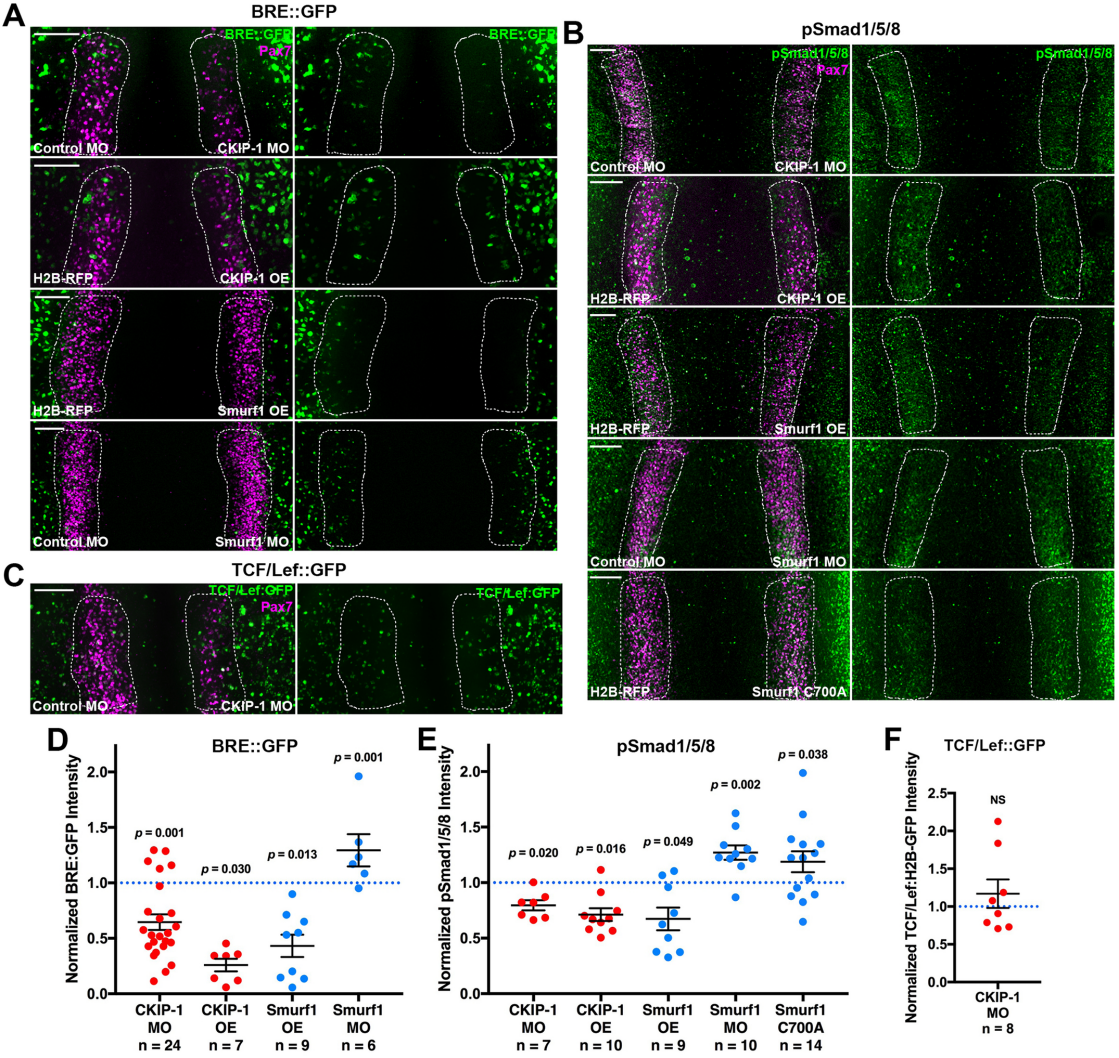
CKIP-1 and Smurf1 regulate BMP signaling and activation of Smad1/5/8. (A) A BRE::GFP reporter construct was coelectroporated with indicated MOs or OE constructs. Embryos then were immunostained at HH7 for Pax7 and GFP. (B) Gastrulating embryos were electroporated with the indicated reagents and then immunostained at HH7 for Pax7 and activated pSmad1/5/8. (C) Embryos were electroporated with a TCF/Lef-driven H2B-GFP reporter construct along with control and CKIP-1 MOs and then immunostained for Pax7 and GFP at HH7. Scale bars represent 100 μm. (D-F) Fluorescence intensity for BRE::GFP (D), pSmad1/5/8 (E), and TCF/Lef::GFP (F) was quantified within the NPB (dashed white lines), normalized to the control side, and displayed with mean ± SEM. Underlying data can be found in S1 Data. *P* values from two-tailed Student *t* test. BMP, bone morphogenetic protein; BRE::GFP, BMP responsive element–driven GFP; CKIP-1, casein kinase interacting protein 1; GFP, green fluorescent protein; H2B, histone 2B; HH, Hamburger-Hamilton stage; MO, morpholino oligonucleotide; OE, overexpression; NPB, neural plate border; NS, not significant; OE, overexpression; Pax7, paired box 7; pSmad1/5/8; phospho-Smads 1/5/8; RFP, red fluorescent protein; Smurf1, Smad ubiquitin regulatory factor 1; TCF/Lef, T-cell factor/lymphoid enhancer factor.

The above results show that CKIP-1 is required during neural crest induction. As BMP signaling is involved in chick neural plate border induction [8,19,20,27], and CKIP-1 mediates BMP signaling in other contexts [29–31], we next asked whether CKIP-1 loss affects BMP signaling output. A BRE::GFP reporter construct [39] revealed that CKIP-1 morphants displayed reduced BMP reporter expression in the neural plate border at HH7 compared to the control side of the same embryo (Fig 4A and 4D). Furthermore, pSmad1/5/8 staining intensity was diminished in the neural plate border of CKIP-1 morphants (Fig 4B and 4E). Interestingly, CKIP-1 OE similarly depletes BRE::GFP and pSmad1/5/8 staining (Fig 4). These results indicate that precise levels of CKIP-1 are required for normal BMP signaling in the neural plate border upstream of pSmad1/5/8. In contrast to BMP signaling, CKIP-1 loss had no effect on expression of a canonical Wnt signaling reporter [47] (Fig 4C and 4F), suggesting that CKIP-1 is dispensable for canonical Wnt signaling.

In osteoblasts, CKIP-1 acts through the SMAD ubiquitination factor Smurf1 to mediate Smad-dependent BMP signaling [30,31]. As our results showed that Smurf1 was also expressed at the neural plate border (Fig 1), we next tested the effect of Smurf1 OE on BMP reporter output. The results show that BMP output was significantly reduced in the neural plate border upon Smurf1 OE (Fig 4A and 4D). Ectopic Smurf1 also diminished pSmad1/5/8 staining intensity (Fig 4B and 4E). We next generated a catalytically inactive Smurf1 construct harboring a cysteine-to-alanine mutation (Smurf1 C700A) previously shown to have a dominant-negative effect on pSmad1/5/8 in both cell lines and in *Xenopus* embryos [34,48,49]. In contrast to suppressed pSmad1/5/8 staining following Smurf1 OE, dominant-negative Smurf1 elevated pSmad1/5/8 staining in the neural plate border (Fig 4B and 4E), consistent with results in *Xenopus* embryos [34]. Similarly, Smurf1 loss of function via MO knockdown also elevated pSmad1/5/8 staining (Fig 4B and 4E), validating the dominant-negative effects of Smurf1 C700A. Together, our results suggest a model in which CKIP-1 and Smurf1 act together to modulate BMP signaling at the neural plate border.

### Smurf1 targets receptor Smads during chick gastrulation

**Fig 5:**
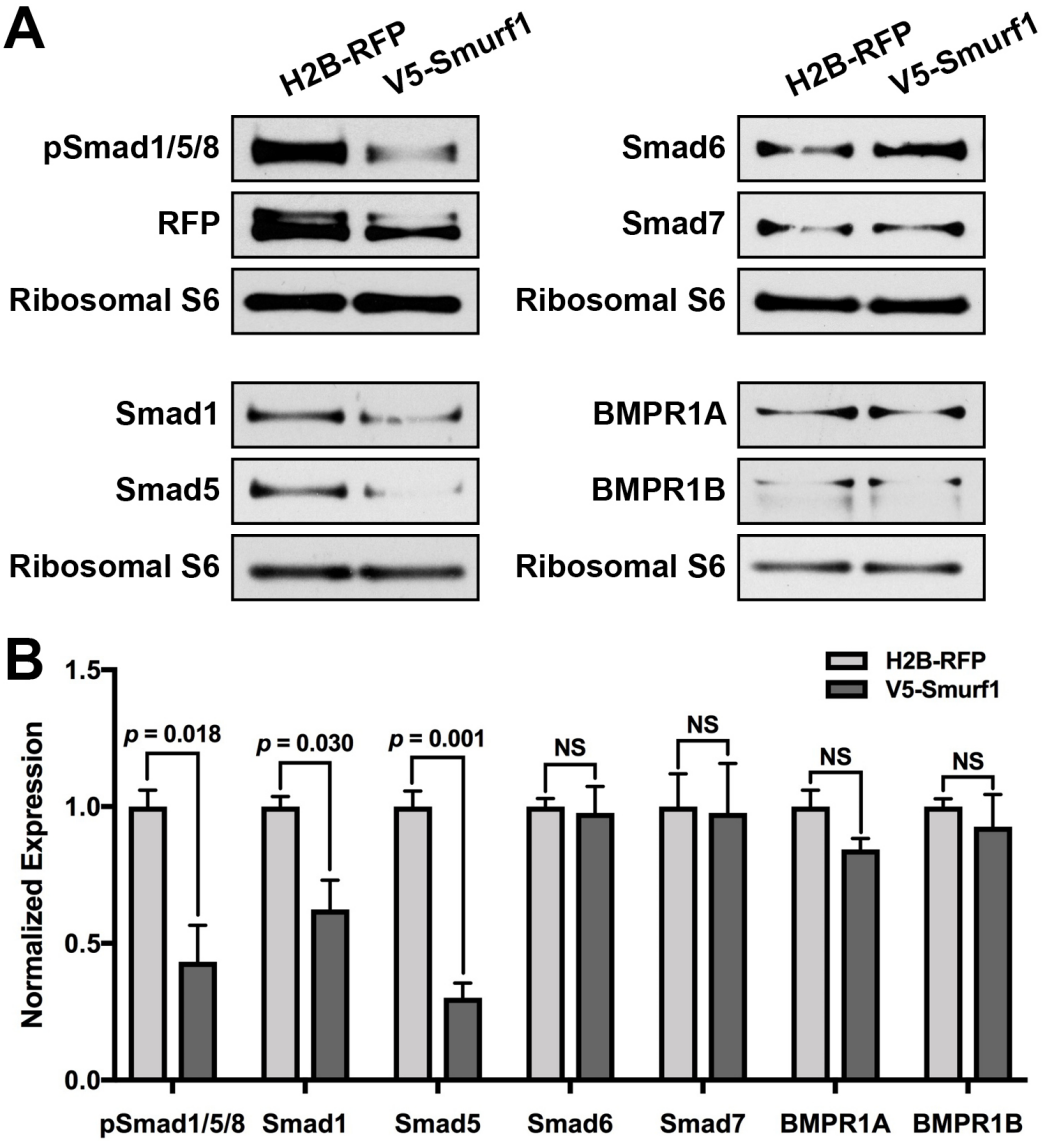
Smurf1 promotes receptor Smad degradation during chick gastrulation. (A) Gastrulating embryos were electroporated with H2B-RFP or V5-Smurf1; then, whole-embryo lysates were prepared at HH7. Ten μg total lysate was loaded per lane, and the resulting blots were processed for the indicated targets, with ribosomal S6 as a loading control. (B) Quantitation of protein expression normalized to ribosomal S6 levels showing means ± SEMs. Underlying data can be found in S1 Data. *P* values from two-tailed Student *t* test. BMPR1A, bone morphogenetic protein receptor type 1A; BMPR1B, bone morphogenetic receptor type 1B; H2B, histone 2B; NS, not significant; pSmad1/5/8, phospho-Smads 1/5/8; RFP, red fluorescent protein; Smurf1, Smad ubiquitin regulatory factor 1.

Smurf1 targets different components of the BMP signaling pathway for proteasomal degradation upstream of Smad phosphorylation, including the BMP type I receptors [50–52], the receptor-activated Smads 1 and 5 [31,48,53], and the inhibitory Smads 6 and 7 [50–52]. To determine which specific molecules are targeted by Smurf1 during chick neural crest induction, we performed western blot analysis on control and V5-tagged Smurf1-overexpressing embryos. As expected, V5-Smurf1 OE resulted in decreased pSmad1/5/8 levels (Fig 5). While we observed comparable reductions in the levels of receptor Smads, there was no effect on the BMP type I receptors nor on the inhibitory Smads (Fig 5). We observe a similar depletion in pSmad1/5/8 and Smad1 protein levels upon CKIP-1 FLAG OE (S5 Fig), consistent with CKIP-1 and Smurf1 each acting on BMP signaling. These results suggest that Smurf1 targets the receptor Smads in HH7 chick embryos, leading to a decrease in phosphorylated Smad1/5/8 and decreased BMP signaling output. Interestingly, Smurf1 promotes BMP inhibition by translocating the inhibitory Smads from the nucleus to the plasma membrane, where Smad6/7 promotes interactions between Smurf1 and the BMP receptors and Smad1/5 [50–52]. The end result of this interaction is proteasomal degradation of the Smurf1 targets and of the inhibitory Smad partners. Since the expression of Smad6/7 appears unaffected by Smurf1 OE, the primary mechanism of action in this context is not likely to involve Smad6/7 function; however, we cannot exclude the possibility that Smad6/7 localization partially contributes to Smurf1 function.

### CKIP-1 promotes Smurf1 degradation at the cell membrane

**Fig 6:**
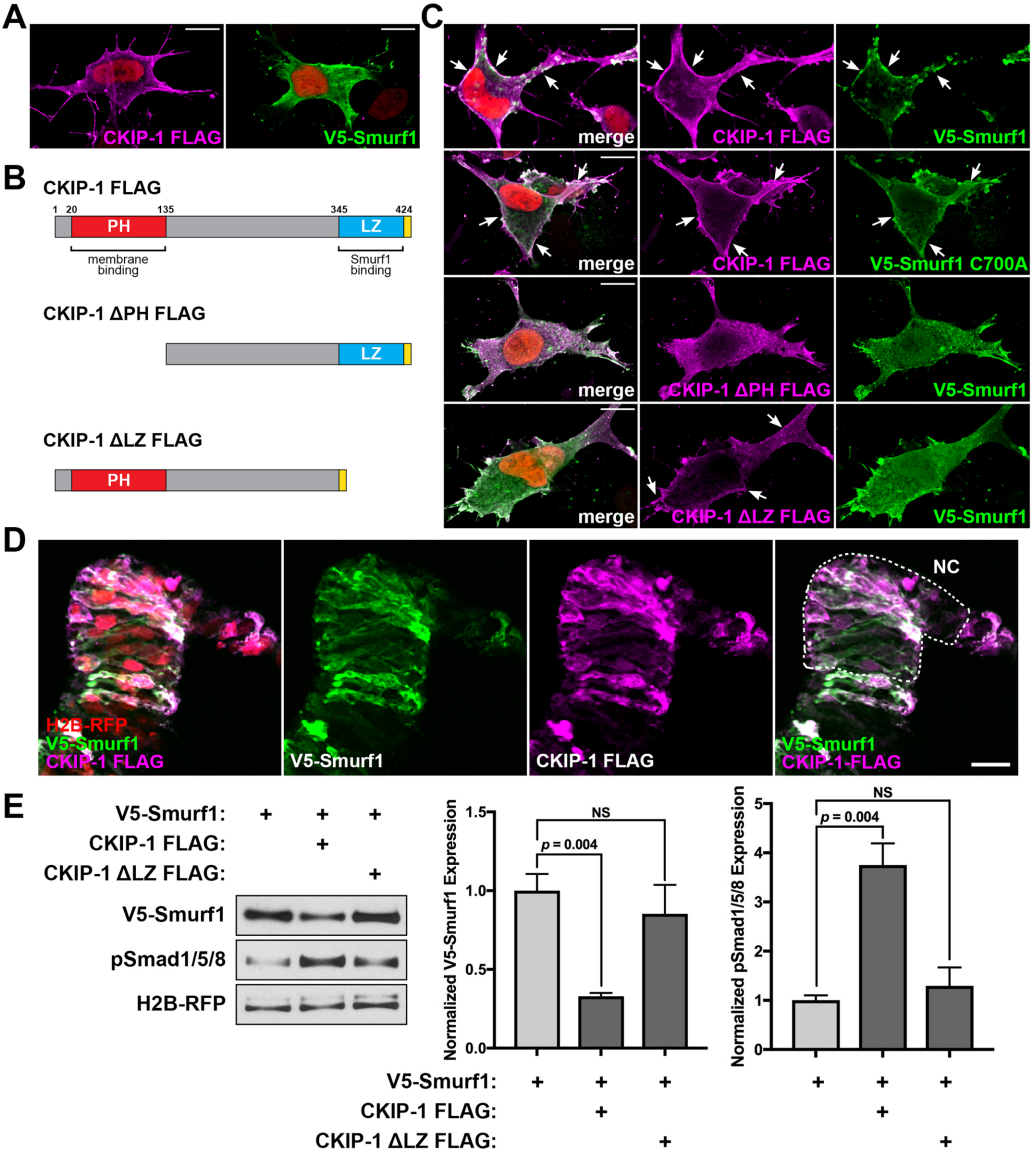
CKIP-1 directly interacts with Smurf1 to promote Smurf1 degradation. DF-1 chicken fibroblasts were transfected with the indicated constructs and then immunostained using anti-FLAG and anti-V5 antibodies. (A) Localization of FLAG-tagged CKIP-1 and V5-tagged Smurf1 alone. (B) Schematics for CKIP-1 FLAG constructs highlighting the membrane-binding PH domain (red) and Smurf1-binding LZ (blue) followed by a C-terminal FLAG tag (yellow). (C) Full-length CKIP-1 FLAG was cotransfected with full-length V5-Smurf1 or V5-Smurf1 C700A dominant-negative mutant. In addition, CKIP-1 FLAG truncation mutants were cotransfected with full-length V5-Smurf1, showing that the PH domain is required for efficient membrane localization, and the LZ domain is required for Smurf1 colocalization. Arrows indicate membrane localization. Scale bars represent 10 μm. (D) Chick embryos were electroporated at HH4 with V5-Smurf1 and CKIP-1 FLAG and then stained for V5 or FLAG at HH8 and imaged in cryosection. Scale bars represent 25 μm. (E) Western blot analysis of HH7 whole-embryo lysates electroporated with constructs indicated and probed for V5, pSmad1/5/8, and RFP. Bar graphs represent normalized V5 and pSmad1/5/8 staining intensity means ± SEM from 3 replicates. Underlying data can be found in S1 Data. *P* values from two-tailed Student *t* test. CKIP-1, casein kinase interacting protein 1; HH, Hamburger-Hamilton stage; LZ, leucine zipper motif; NC; neural crest domain; NS, not significant; PH, pleckstrin homology; pSmad1/5/8, phospho-Smads 1/5/8; RFP, red fluorescent protein; Smurf1, Smad ubiquitin regulatory factor 1.

Since we observed a role for CKIP-1 and Smurf1 in Smad levels and the BMP signaling response, and previous work has shown a direct interaction between CKIP-1 and Smurf1 upstream of Smad stability [31,32], we next asked if CKIP-1 and Smurf1 colocalize. To this end, we tested the possibility of such a physical interaction using chicken DF-1 fibroblasts and neurulating chick embryos. In DF-1 cells, transfection of CKIP-1 FLAG alone showed CKIP-1 localization to the cell membrane, while V5-Smurf1 transfection alone showed localization to the cytoplasm (Fig 6A). When cotransfected, CKIP-1 FLAG and V5-Smurf1 colocalized at the cell membrane (Fig 6C), suggesting a direct interaction between these two proteins. Similarly, dominant-negative V5-Smurf1 C700A colocalized with CKIP-1 FLAG at the membrane (Fig 6C), indicating that Smurf1 does not require catalytic function to interact with CKIP-1. Finally, we employed two CKIP-1 FLAG mutants that lack either the membrane-binding pleckstrin homology domain (ΔPH) or the Smurf1-binding leucine zipper domain (ΔLZ, Fig 6C). When membrane binding was lost, both CKIP-1 FLAG and V5-Smurf1 colocalized in the cytoplasm. However, when the putative Smurf1-binding domain was deleted, V5-Smurf1 localized to the cytoplasm, while CKIP-1 FLAG remained membrane bound. We next tested if CKIP-1 FLAG and V5-Smurf1 colocalize in neural crest cell membranes. We observed that coelectroporation of both constructs results in strong membrane localization in vivo at HH8 (Fig 6D), consistent with cell culture experiments. Together, these results suggest that CKIP-1 and Smurf1 physically interact at the cell membrane.

Since CKIP-1 enhances Smurf1 activity, and Smurf1 is a target of its own ubiquitination [31], we asked if CKIP-1 promotes Smurf1 autodegradation during neural crest induction. To test this, we performed western blotting using electroporated chicken embryos at HH7 (Fig 6E). Addition of CKIP-1 FLAG depleted V5-Smurf1 levels and resulted in a corresponding increase in pSmad1/5/8. This effect was lost when V5-Smurf1 was expressed with CKIP-1 FLAG ΔLZ, the truncation mutation that abolishes direct binding to the WW domains in Smurf1 [31]. These results indicate that direct interactions between CKIP-1 and Smurf1 promote Smurf1 autodegradation, suggesting that CKIP-1 modulates Smurf1 activity to maintain neural crest induction.

### Epistasis experiments indicate that CKIP-1 inhibits Smurf1 in the neural plate border

**Fig 7:**
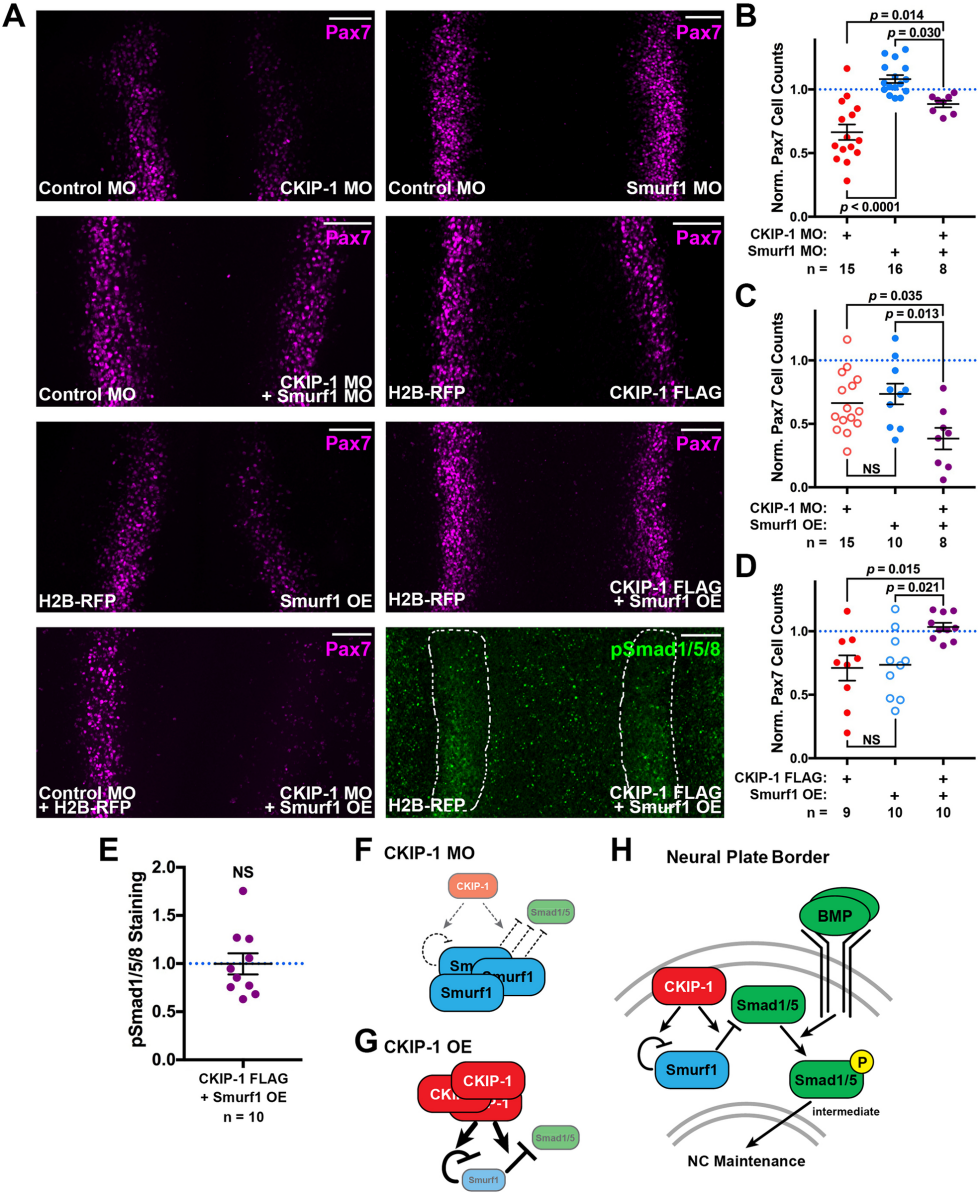
CKIP-1 negatively regulates Smurf1 activity during NPB induction. (A) HH4 embryos were electroporated with loss- or gain-of-function constructs indicated and then immunostained for Pax7 or pSmad1/5/8 at HH7. Scale bars represent 100 μm. (B-D) Normalized quantitation of Pax7 cell counts following epistasis perturbations as described in A, with *P* values from one-way ANOVA with Tukey’s post hoc test. (E) pSmad1/5/8 signal intensity was quantitated within the NPB, normalized to the control side, and displayed with mean ± SEM. Underlying data can be found in S1 Data. *P* values from two-tailed Student *t* test. (F-H) Schematic model for intracellular regulation of BMP signaling by CKIP-1/Smurf1 in CKIP-1 loss of function (F), CKIP-1 OE (G), and wild-type (H) contexts. By modulating the activity of Smurf1, CKIP-1 establishes a balance between Smurf1 and Smad1/5 degradation to permit intermediate levels of BMP-mediated Smad activation, thereby maintaining expression of NPB genes, including Pax7, during NPB induction. BMP, bone morphogenetic protein; CKIP-1, casein kinase interacting protein; H2B, histone 2B; HH, Hamburger-Hamilton stage; MO, morpholino oligonucleotide; NC, neural crest; NPB, neural plate border; NS, not significant; OE, overexpression; Pax7, paired box 7; pSmad1/5/8, phospho-Smads 1/5/8; RFP, red fluorescent protein; Smurf1, Smad ubiquitin regulatory factor 1.

Finally, we examined genetic interactions between CKIP-1 and Smurf1 during neural plate border induction. First, we performed MO-mediated Smurf1 knockdown and observed a mild but significant increase in Pax7+ neural plate border cells (Fig 7A and 7B; Student *t* test, *p* = 0.037). Since this effect is opposite to CKIP-1 MO–mediated Pax7 loss, we performed epistasis analysis by combining the two MOs, reasoning that if CKIP-1 and Smurf1 act in a genetic pathway, the downstream phenotype should prevail. Combined MOs restored Pax7 cell counts to a level more similar to Smurf1 MO than CKIP-1 MO (Fig 7A and 7B), consistent with Smurf1 acting downstream of CKIP-1.

Furthermore, Smurf1 OE alone resulted in Pax7 reduction at HH7, comparable to CKIP-1 knockdown (Fig 7A and 7C) and consistent with the reduced BMP signaling observed in each condition (Fig 4). If CKIP-1 acts to inhibit Smurf1, CKIP-1 loss paired with Smurf1 gain is predicted to have synergistic effects on BMP signaling and result in a stronger loss of Pax7. As expected, coelectroporation of CKIP-1 MO with Smurf1 OE caused a more dramatic loss of Pax7 than either reagent alone (Fig 7A and 7C). These results are consistent with a model in which CKIP-1 functions to inhibit Smurf1 upstream of neural plate border induction.

A prediction of this model is that CKIP-1 OE should counteract the effects of Smurf1 OE, effectively rescuing neural crest formation. Indeed, the results show that OE of Smurf1 combined with CKIP-1 FLAG restored the numbers of Pax7-expressing cells to the level observed in control embryos (Fig 7A and 7D). Furthermore, Smurf1 OE combined with CKIP-1 FLAG OE resulted in normal levels of pSmad1/5/8 staining (Fig 7A and 7D), demonstrating the epistatic relationship between Smurf1 and CKIP-1 upstream of pSmad1/5/8. This result is consistent with the effects on pSmad1/5/8 expression when comparing Smurf1 OE to combined Smurf1 and CKIP-1 OE by western blotting (Fig 6E).

Taken together, these results suggest that the combined actions of and balance between BMP4, CKIP-1, and Smurf1 are critical for determining the levels of BMP signaling at the neural plate border. Whereas high BMP4 leads to high pSmad1/5/8 levels in the nonneural ectoderm, high levels of Smurf1 attenuate BMP signaling by causing degradation of receptor Smads. At the neural plate border, there is overlapping expression of CKIP-1, Smurf1, and BMP4. By promoting Smurf1 activity, CKIP-1 establishes a balance between Smurf1 autodegradation and Smad1/5 degradation to achieve intermediate pSmad1/5/8 levels at the neural plate border. Upon CKIP-1 loss of function, Smurf1 activity is reduced, resulting in less autodegradation and ultimately elevation of Smurf1 protein levels; this heightened Smurf1 level then promotes Smad1/5 degradation (Fig 7G). Conversely, CKIP-1 OE enhances Smurf1 activity; Smurf1 ubiquitinates itself and Smad1/5, resulting in degradation of both targets (Fig 7H). Thus, the balance of CKIP-1, Smurf1, and receptor Smads in neural plate border cells results in the appropriate levels of BMP signaling required for neural plate border maintenance (Fig 7F).

## Discussion

In this study, we demonstrate that CKIP-1 is required for BMP signaling upstream of Smad activation during gastrulation and for subsequent neural crest induction in the chick neural plate border. Furthermore, we show that Smurf1 function suppresses BMP signaling by diminishing pSmad1/5/8 at the level of the receptor Smads, thus suppressing neural crest induction. Together, our data are consistent with a model in which BMP signals are attenuated intracellularly in the chick neural plate border, at least in part via CKIP-1 and Smurf1, resulting in intermediate pSmad1/5/8 levels. These intermediate pSmad1/5/8 levels in turn contribute to neural crest induction via maintenance of expression of Pax7 and other genes of the neural plate border gene regulatory module (Fig 7H). While Smurf1 acts to suppress BMP-dependent Smad levels via ubiquitination [30,31], CKIP-1 acts to establish intermediate pSmad1/5/8 levels by mediating the balance between Smurf1 autodegradation and Smad1/5 targeting (Fig 7H). Interestingly, this relationship has the opposite effect of that observed in osteoblasts, in which CKIP-1 loss reduces Smurf1-mediated Smad degradation [30,33]. Our data suggest a direct interaction between CKIP-1 and Smurf1 at the plasma membrane, which may facilitate Smad degradation by promoting localization of Smurf1 to the site of receptor-mediated Smad phosphorylation.

In *Xenopus*, a two-step model for neural crest induction has been proposed in which Wnt activation and BMP inhibition, mediated by mesodermal signals from the dorsolateral marginal zone, are followed by simultaneous Wnt and BMP activation by the intermediate mesoderm to induce presumptive neural crest [46,54]. Work in chick supports this two-step model, wherein early Wnt signaling acts to induce BMP expression in neural plate border cells, followed by activation of both pathways during the later maintenance step [27]. On the other hand, there has been controversy in the literature regarding a morphogen model in which intermediate BMP signaling levels are proposed to induce the neural crest, with high levels of BMP signaling inducing epidermal fates. While this idea has been supported by data in *Xenopus* and zebrafish embryos [15–17,55], data in the chick raised questions as to its validity [19,23]. However, our data showing intermediate pSmad1/5/8 levels at the neural plate border are consistent with previous indications in the literature [8,38] to suggest that intermediate BMP signaling does occur in the chick neural plate border.

In *Xenopus* and zebrafish, experimental BMP signaling modulation shifts the location of the neural plate border at the expense of the neural plate or nonneural ectoderm [15,18,55–57]. Consistent with this, misexpression of BMP4 or its secreted inhibitors by recombinant protein-soaked beads shifts the location of the neural plate border [19]. In our study, we do not observe this effect, but rather we affect the number of Pax7-expressing cells without obviously changing the overall size of the neural plate border. This is likely because CKIP-1 and Smurf1 act on maintenance of neural crest induction after the ectodermal territories have been patterned. Our results uncover “fine-tuning” of BMP signaling within individual cells, thereby biasing their fates in response to BMP signaling. Since neural plate border cells normally coexpress cell markers characteristic of the disparate fates that can arise from the neural plate border—like Pax7, Sry-related HMg-Box gene 2 (Sox2), and Six1 [58]—we speculate that fine-scale, cell-autonomous modulation of signaling output levels helps promote certain transcriptional programs at the expense of others to refine the neural plate border cells toward their definitive cell fates.

BMP signaling modulation has been observed at multiple levels. The expression of numerous ligands allows for the formation of multiple ligand hetero- and homodimers that may influence signaling strength [59,60]. Notably, in addition to BMP4, BMP7 also appears to be expressed in the neural plate border [22,23], and BMP4/7 heterodimers are strong activators of the BMP receptors [60,61]. Thus, intracellular regulation becomes essential in the neural plate border to attenuate BMP reception. In addition to its role in promoting Smad1/5/8 degradation, Smurf1 targets tumor necrosis factor receptor–associated factor 4 (TRAF4) protein for degradation by ubiquitination [62]. Interestingly, TRAF4 has been implicated in *Xenopus* neural plate border induction by potentiating TGF-β signaling [62], suggesting that Smurf1 dampens BMP signaling at more than just the level of Smads.

The neuralizing activity of Smurf1 appears to be conserved between *Xenopus* and chick, since XtSmurf1 OE diminishes pSmad1/5/8 levels, resulting in expansion of neural tissue, whereas its loss expands epidermal markers [32,34]. While our results are consistent with those in *Xenopus*, CKIP-1- and Smurf1-null mice are viable and fertile [31,63]. Since Smurf1 loss in *Xenopus* studies shows presumably lethal embryonic patterning defects [32,34], we suspect that normal development in knockout mice reflects genetic redundancy or compensation in these transgenic models that is not provoked by transient perturbations. One possible compensatory mechanism may be through the function of the closely related ubiquitin ligase Smad ubiquitin regulatory factor 2 (Smurf2). Smurf2 is not strongly expressed in chicken neural crest [35]. However, Smurf2 expression is comparable to that of Smurf1 in mouse neural crest cells [64], suggesting that Smurf2 may compensate for loss of CKIP-1 or Smurf1 in mice.

Based on our data, we propose the following possible model. In chick, Smurf1 acts to suppress BMP signaling by targeting receptor Smads for degradation. CKIP-1 acts to attenuate this via modulating Smurf1 function. OE of CKIP-1 enhances the activity of Smurf1, resulting in the targeting of both Smads and Smurf1 for degradation and thus depleted pSmad1/5/8 levels and reduced Pax7 expression. Conversely, CKIP-1 loss of function relieves autodegradation of Smurf1, resulting in increased Smurf1 levels and similarly depleted BMP output and Pax7 activation. At their endogenous levels, these proteins together allow for the intermediate BMP activation in the neural plate border that is a prerequisite for neural crest maintenance.

Taking the present results together with previous literature helps to formulate a refined timeline of neural crest induction in chick and to resolve the respective roles of FGF, BMP, and Wnt signaling therein. First, FGF signaling may prime the neural plate border through extracellular signal-regulated kinase 1/2 (Erk1/2) activity to respond to BMP-inductive signals [8]. This occurs at early gastrula stages and is no longer required after BMP signaling begins [8]. Wnt signaling in this initial phase of induction acts to induce BMP expression [27], in keeping with observations in *Xenopus* [46,55]. Next, intermediate BMP signaling, together with Wnt signaling, is required for robust Pax7 expression, while strong BMP signaling promotes epidermal fates [27]. Our data suggest that BMP signaling is modulated, in part, by intracellular CKIP-1 and Smurf1 in chick. While our results indicate that CKIP-1 and Smurf1 function prior to HH7, these genes are expressed during gastrulation and prior to experimental accessibility; thus, we cannot exclude the possibility that they are functionally relevant at earlier stages. Finally, our previous results have shown that subsequent neural crest specification is activated by the Wnt1-dependent effector axin up-regulated 1 (Axud1), which complexes with neural plate border specifiers Pax7 and Msx1 to drive neural crest specification [9]. Interestingly, Axud1 expression initiates at HH7, just when our results show that CKIP-1 loss no longer affects neural crest formation. Taken together, these combined results suggest sequential rather than concomitant roles for FGF and BMP in neural crest induction, with multiple roles for Wnt signaling during this phase. This also supports the two-step model for neural crest induction [27,46], with intermediate BMP signaling contributing to the maintenance step in the neural plate border.

## Materials and methods

### Ethics statement

The Office of Laboratory Animal Welfare (OLAW) of the National Institutes of Health (NIH) adheres to the Public Health Service (PHS) Policy on the Humane Care and Use of Laboratory Animals, under which prehatching avian embryos are not considered live vertebrate animals. As such, no animal approvals were required during the course of this study.

### Embryos and perturbations

Fertilized chicken eggs were acquired from McIntyre Poultry & Eggs (Lakeside, CA) and AA Lab Eggs (Westminster, CA) and incubated at 39 °C to the desired HH stage [65]. Electroporations were performed *ex ovo* as previously described [66] using five 5.2 V pulses for 50 ms at 100 ms intervals and then cultured in albumin with 1% added penicillin/streptomycin (Corning cellgro). All embryos used for analysis and presentation were selected for high electroporation efficiency as determined by FITC signal for MO and RFP signal for OE electroporations. Examples showing electroporation efficiency are presented in Figs 2 and 3B, S3 and S4 Figs. FITC-labeled MOs (Gene Tools) include random control MO (5′–CCTCTTACCTCAGTTACAATTTATA–3′), translation-blocking CKIP-1 MO (5′–TGGCTGAATTATTCTTTTTCATCGT–3′), and Smurf1 MO (5′–TCGCGCCCTGCGGCGAGATCAAC–3′), which were injected between 0.6 and 1.5 mM together with 1 μg/μl pBluescript carrier DNA to improve transfection efficiency. OE constructs were injected similarly at the indicated concentrations. Stained embryos were prepared for cryosectioning following incubations in 5% sucrose (30 minutes at room temperature), 15% sucrose (overnight at 4 °C), then 7.5% gelatin (overnight at 39 °C), and then cryosectioned at a thickness of 16 μm.

CRISPR-mediated knockouts were performed by coelectroporation of Cas9 protein with targeting gRNAs. A nonbinding control gRNA (5′–GCACTGCTACGATCTACACC–3′) has been described [42], and a CKIP-1-targeting gRNA was designed in the second exon (5′–GCAATCTGCGCAGCCCGAGA–3′) using CHOPCHOP [67]. gRNA templates were generated by PCR using a short oligo method [68], and gRNAs were transcribed using the HiScribe T7 Quick High Yield RNA Synthesis Kit (New England Biolabs). For injection, equal concentrations of Cas9 protein (New England Biolabs) and gRNA were mixed and incubated at 37 °C for 15 minutes before dilution with 10 mM Tris-HCl, pH 8.5 and lineage-marking pCI H2B-RFP plasmid [41] to a final concentration of 0.5 μg/μl each Cas9 and gRNA and 2 μg/μl pCI H2B-RFP. Embryos were then electroporated at HH3 as described above.

### Cloning and constructs

Full-length CKIP-1 (Genbank Accession #KY982274) and Smurf1 (Genbank Accession #KY982275) ORFs were cloned from an HH9–10 cDNA library, using standard molecular procedures. Products were cloned into pGEM T Easy (Promega) for sequencing; these clones were then used as a template for subcloning into an expression vector driven by the CAG promoter and containing a downstream IRES driving H2B-RFP expression [41]. Each expression construct included a 5′ Kozak consensus sequence to promote efficient translation. Sequential PCR amplifications were performed to add a C-terminal triple glycine linker followed by a FLAG tag to the CKIP-1 ORF or to add an N-terminal V5 tag followed by a GGSGG linker and then the Smurf1 ORF. Similarly, the cysteine at position 700 in the chick Smurf1 sequence was mutated to alanine (Smurf1 C700A) by overlap PCR and subcloning. All primer sequences used are presented in S1 Table. TCF/Lef-driven H2B-GFP [47] (Addgene plasmid #32610) and BRE::GFP [39] reporter constructs were generous gifts from Anna-Katerina Hadjantonakis and Elisa Martí, respectively.

### In situ hybridization and HCR

BMP4 and Msx1 in situ probes were generated as previously described [36,45]. For probe synthesis, 823 bp and 982 bp fragments of the CKIP-1 and Smurf1 cDNAs, respectively, were subcloned into pGEM T Easy (Promgea). DIG-labeled (Roche) RNA probes were generated using T7 and SP6 RNA polymerases (Promega), respectively, and then purified using Illustra ProbeQuant G-50 Micro Columns (GE Healthcare). Hybridization was carried out as previously described [9]. Briefly, embryos were fixed in 4% paraformaldehyde (PFA) at 4 °C overnight and then dehydrated in a methanol series and stored in 100% methanol at −20 °C. Rehydrated embryos were treated with proteinase K (10 μg/mL) to improve tissue penetration, postfixed in 4% PFA with 0.2% glutaraldehyde, and then hybridized with 1 ng/μl probe mixture overnight at 70 °C. After extensive washes, ISH probes were detected using alkaline phosphatase–conjugated anti-DIG Fab fragments (1:2,000; Roche) overnight at 4 °C and developed using a mixture of nitro-blue tetrazolium (NBT, Roche) and 5-bromo-4-chloro-3’-indoylphosphate p-toluidine (BCIP, Roche).

For in situ HCR, a kit containing a DNA probe set, a DNA HCR amplifier, and hybridization, wash, and amplification buffers was purchased from Molecular Instruments (molecularinstruments.org) for each target transcript. The BMP4, CKIP-1, and Smurf1 probes initiate B1 (Alexa488), B3 (Alexa546), and B5 (Alexa647) amplifiers, respectively. In situ HCR was performed using the previously detailed protocol [69].

### Immunohistochemistry

Embryos were fixed for immunostaining for 20 minutes at room temperature in 4% PFA in phosphate buffer for 20 minutes, and all washes and incubations were performed in TBST + Ca^2+^ (50 mM Tris-HCl, 150 mM NaCl, 1 mM CaCl_2_, 0.5% Triton X-100). Embryos were blocked in 10% donkey serum for 2 hours at room temperature; then, primary and secondary antibody incubations occurred in 10% donkey serum for 2 nights at 4 °C. Primary antibodies used include Developmental Studies Hybridoma Bank mouse IgG1 anti-Pax7 (1:10; PAX7) and mouse IgM anti-HNK-1 (1:5; 3H5); Novus Biologicals goat anti-FITC (1:500; NB600-493); Sigma rabbit anti-FLAG (1:100; F7425); Invitrogen Mouse IgG2a anti-V5 (1:500; R960-25); EMD Millipore rabbit anti-phosphohistone H3 (1:500; 06–570) and rabbit anti-Sox9 (1:1,000; AB5535); R&D Systems rabbit anti-cleaved caspase 3 (1:300; AF835) and goat anti-Sox10 (1:100; AF2864); Abcam rabbit anti-GFP (1:500; AB290); and Cell Signaling Technologies rabbit anti-pSmad1/5/8 (1:100; 13820) and rabbit anti-Snai2 (1:500; 9585). Primary antibodies were detected using Alexa Fluor 350/488/568/647-conjugated donkey secondary antibodies (1:500; Molecular Probes).

### Western blotting

Electroporated embryos were cultured to the desired stage and homogenized in 8 M Urea/2.5% SDS followed by 30 minutes at 65 °C and storage at −80 °C. Following manufacturer’s instructions, 10 μg of total protein lysate per lane was diluted and run on 10-well Bolt 4%–12% Bis-Tris Plus gels (Invitrogen). Protein was then transferred to 0.2 μm nitrocellulose (Amersham) in 25 mM Tris/200 mM glycine/20% methanol at 100 V for 1 hour at 4 °C. Blots were washed with TBST pH 7.6 (50 mM Tris, 150 mM NaCl, and 0.1% Tween20) and blocked in 5% BSA/TBST or 5% milk/TBST for 1 hour at room temperature. Primary antibody incubations were carried out overnight at 4 °C. Primary antibodies used include Abcam mouse anti-RFP (1:1,000; ab125244), rabbit anti-Smad6 (1:2,000; ab80049), and rabbit anti-BMPR1B (1:1,000; ab155058); Cell Signaling Technologies rabbit anti-S6 ribosomal protein (1:2,000; 2217) and rabbit anti-pSmad1/5/8 (1:1,000; 13820); Invitrogen mouse anti-V5 (1:5,000; R960-25); MyBioSource rabbit anti-BMPR1A (1:1,000; MBS855162); Novus Biological rabbit anti-CKIP-1 (1:100; NBP1-76238); and Santa Cruz BioTechnology mouse anti-Smad1 (1:300; 913c1b), mouse anti-Smad5 (1:200; YY-6), and mouse anti-Smad7 (1:200; Z8B). Primary antibodies were detected using anti-rabbit- and anti-mouse-HRP conjugated secondary antibodies (KPL, 1:40,000 and 1:20,000, respectively) in 5% milk/TBST for 1 hour at room temperature, followed by chemiluminescent detection (ECL Prime Western Blotting System, GE Healthcare). Resulting bands were quantified using Fiji [70].

### DF-1 cell culture and transfection

Immortalized chicken DF-1 fibroblast cells (ATCC CRL-12203) were cultured at 37 °C in 5% CO_2_ in DMEM (Corning) supplemented with 10% fetal bovine serum (Gibco) and penicillin/streptomycin (Corning). Cells were transfected on 18 mm round coverslips in 12-well plates at 70% confluency using Lipofectamine 3000 (Invitrogen). Transfected cells were incubated for 24 hours and then fixed and processed for immunostaining as described above.

### Imaging, analysis, and statistical methods

Immunostained embryos were imaged in whole mount and in section using a Zeiss Imager.M2 with an ApoTome.2 module, and transfection experiments were imaged using a Zeiss LSM 880 confocal microscope with an AiryScan module. All whole-mount images display maximum intensity projections of Z-stacks. Cell counting was performed using the Analyze Particles feature on binarized images in Fiji [70]. Fluorescence intensity was determined within manually drawn regions of interest by measuring integrated density of background-subtracted images. Experimental values were normalized to the contralateral control side. Statistical tests performed include paired two-tailed Student *t* test when comparing two measurements within a single embryo, unpaired two-tailed Student *t* tests when comparing independent groups of embryos, and one-way ANOVA with Tukey’s post hoc analysis for multiple comparisons. For all statistical tests, at least 2 independent experiments were performed. All *t* tests passed a power analysis in G*Power 3.1 [71] with a power cutoff of 0.80.

## Supporting information

S1 Fig Expression of *bmp4*, *ckip-1*, and *smurf1* highlight a complex relationship with BMP pathway activation. (A) Whole-mount in situ hybridization shows expression of *bmp4*, *ckip-1*, and *smurf1* in wild-type HH6 chick embryos. (B) HCR showing expression of *bmp4*, *ckip-1*, and *smurf1* in wild-type HH5 chick embryos. (C) Negative control shows minimal background following HCR protocol. (D) Gastrulating embryos were electroporated with the BRE::GFP reporter (cyan) and then immunostained for pSmad1/5/8 and Pax7. Line traces of staining intensities within the boxed region show intermediate BMP pathway activation in the neural plate border. Underlying data can be found in S1 Data. Scale bars represent 200 μm. BMP, bone morphogenetic protein; BRE::GFP, BMP responsive element–driven green fluorescent protein; HCR, hybridization chain reaction; HH, Hamburger-Hamilton stage; NNE, non-neural ectoderm; NP, neural plate; NPB, neural plate border; Pax7, paired box 7; pSmad1/5/8, phospho-Smads 1/5/8.
(TIF)

S2 Fig Chicken *ckip1* is expressed in the NPB and in the premigratory and migratory neural crest. In situ hybridization was performed on wild-type chicken embryos at the indicated HH and are presented in whole-mount dorsal views (A) and transverse sections (B). HNK-1 immunostaining labels migrating NCs in sections. Arrows indicate premigratory NC (HH9) and migratory NC (HH10 and HH13). HH, Hamburger-Hamilton stage; HNK-1, human natural killer 1; NC, neural crest; NP, neural plate; NPB, neural plate border.
(TIF)

S3 Fig CKIP-1 loss of function provokes loss of neural crest. (A) Control MO and CKIP-1 MO electroporated embryos shown at HH10 display ranges of neural crest loss indicated in each panel, as determined by Pax7 immunostaining. Insets show corresponding FITC-conjugated MO electroporation. (B) Western blotting analysis of embryos electroporated with control MO, CKIP-1 MO, or CKIP-1 FLAG and blotted for CKIP-1 and ribosomal S6. The CKIP-1 FLAG lane was loaded with 0.5 μg whole-embryo lysate, while 10 μg was loaded for the other lanes. CKIP-1 intensity was normalized to ribosomal S6 as a loading control; displayed are means ± SEMs. (C) Bilateral electroporations were performed at HH4 with a control mixture (left; control MO and pCI-H2B-RFP) and an experimental mixture (right; doses indicated in D). Resulting embryos were cultured to HH10 and analyzed for Pax7 expression. (D) Quantitation of Pax7 reduction from rescue experiments. (E) Dorsal views of CKIP-1 MO alone (MO at 0.7 mM, H2B-RFP at 2 μg/μl), rescue (CKIP-1 MO at 0.7 mM, CKIP-1 FLAG at 1.5 μg/μl), and CKIP-1 FLAG alone (Control MO at 0.7 mM, CKIP-1 FLAG at 2 μg/μl) embryos displaying Pax7, CKIP-1 FLAG, FITC, and H2B-RFP. Displayed are cropped images as outlined in C. White arrow indicates rescued neural crest cell formation. (F) Embryos were coelectroporated at HH3 with Cas9 protein complexed with nonbinding control or CKIP-1-targeting gRNAs and H2B-RFP as a lineage label. Resulting embryos were incubated to HH9 and processed for Pax7 and Sox9 immunostaining. Cell counts quantitated from whole-mount images demonstrate that CRISPR/Cas9-mediated CKIP-1 knockout phenocopies neural crest loss observed with CKIP-1 MO. Displayed are cell counts normalized to the control side with mean ± SEM. *P* values from two-tailed Student *t* test. Scale bars represent 100 μm. Underlying data can be found in S1 Data. Cas9, CRISPR-associated protein 9; CKIP-1, casein kinase interacting protein 1; CRISPR, clustered regularly interspaced short palindromic repeat; FITC, fluorescein isothiocyanate; gRNA, guide RNA; H2B, histone 2B; HH, Hamburger-Hamilton stage; MO, morpholino oligonucleotide; Pax7, paired box 7; RFP, red fluorescent protein; Sox9, Sry-related HMg-Box gene 9; Sox10, Sry-related HMg-Box gene 10.
(TIF)

S4 Fig CKIP-1 loss does not affect cell proliferation or cell survival. (A,B) CKIP-1 MO–electroporated embryos were harvested at HH7 and then immunostained for Pax7, FITC, and phospho-histone H3 (A) or cleaved-caspase 3 (B). (C) Regions of interest were drawn around the neural plate border (dashed white lines), and phospho-histone H3 cell counts or cleaved-caspase 3 staining intensity was measured and normalized to control and displayed with means ± SEMs. Underlying data can be found in S1 Data. *P* values from two-tailed Student *t* test. Scale bars represent 100 μm. CKIP-1, casein kinase interacting protein 1; FITC, fluorescein isothiocyanate; HH, Hamburger-Hamilton stage; MO, morpholino oligonucleotide; NS, not significant; Pax7, paired box 7.
(TIF)

S5 Fig CKIP-1 FLAG suppresses pSmad1/5/8 and Smad1 levels. (A) Gastrulating embryos were electroporated with H2B-RFP or CKIP-1 FLAG; then, whole-embryo lysates were prepared at HH7. Ten μg total lysate was loaded per lane, and the resulting blots were processed for the indicated targets, with ribosomal S6 as a loading control. (B) Quantitation of protein expression normalized to ribosomal S6 levels showing means ± SEMs. Underlying data can be found in S1 Data. *P* values from two-tailed Student *t* test. CKIP-1, casein kinase interacting protein 1; H2B, histone 2B; HH, Hamburger-Hamilton stage; pSmad1/5/8, phospho-Smads 1/5/8; RFP, red fluorescent protein.
(TIF)

S1 Data Underlying data for all charts. (XLSX)

S1 Table Primer sequences used in this study. (XLSX)

## Abbreviations

Axud1: axin upregulated 1
BCIP: 5-bromo-4-chloro-3’-indoylphosphate p-toluidine
BMP: bone morphogenetic protein
BRE::GFP: BMP responsive element–driven GFP
Cas9: CRISPR-associated protein 9
CRISPR: clustered regularly interspaced short palindromic repeat
Erk1/2: extracellular signal-regulated kinase 1/2
FGF: fibroblast growth factor
FITC: fluorescein isothiocyanate
FoxD3: forkhead box D3
gRNA: guide RNA
H2B: histone 2B
HCR: hybridization chain reaction
HH: Hamburger-Hamilton stage
HNK-1: human natural killer 1
IRES: internal ribosome entry site
LZ: leucine zipper domain
MO: morpholino oligonucleotide
Msx1: msh homeobox 1
NBT: nitro-blue tetrazolium
NIH: National Institutes of Health
OE: overexpression
OLAW: Office of Laboratory Animal Welfare
ORF: open reading frame
Pax7: paired box 7
PFA: paraformaldehyde
PH: pleckstrin homology domain
PHS: Public Health Service
pSmad1/5/8: phospho-Smad 1/5/8
RFP: red fluorescent protein
Smurf1: Smad ubiquitin regulatory factor 1
Smurf2: Smad ubiquitin regulatory factor 2
Sox10: Sry-related HMg-Box gene 10
Sox2: Sry-related HMg-Box gene 2
Sox9: Sry-related HMg-Box gene 9
TGF-β: transforming growth factor beta
TRAF4: tumor necrosis factor receptor–associated factor 4
