# Supplementary figures and images for: Intracellular attenuation of BMP signaling via CKIP-1/Smurf1 is essential during neural crest induction
Source: PLoS Biol. 2018 Jun 27;16(6):e2004425. doi: 10.1371/journal.pbio.2004425 (PMC6039030; doi:10.1371/journal.pbio.2004425)

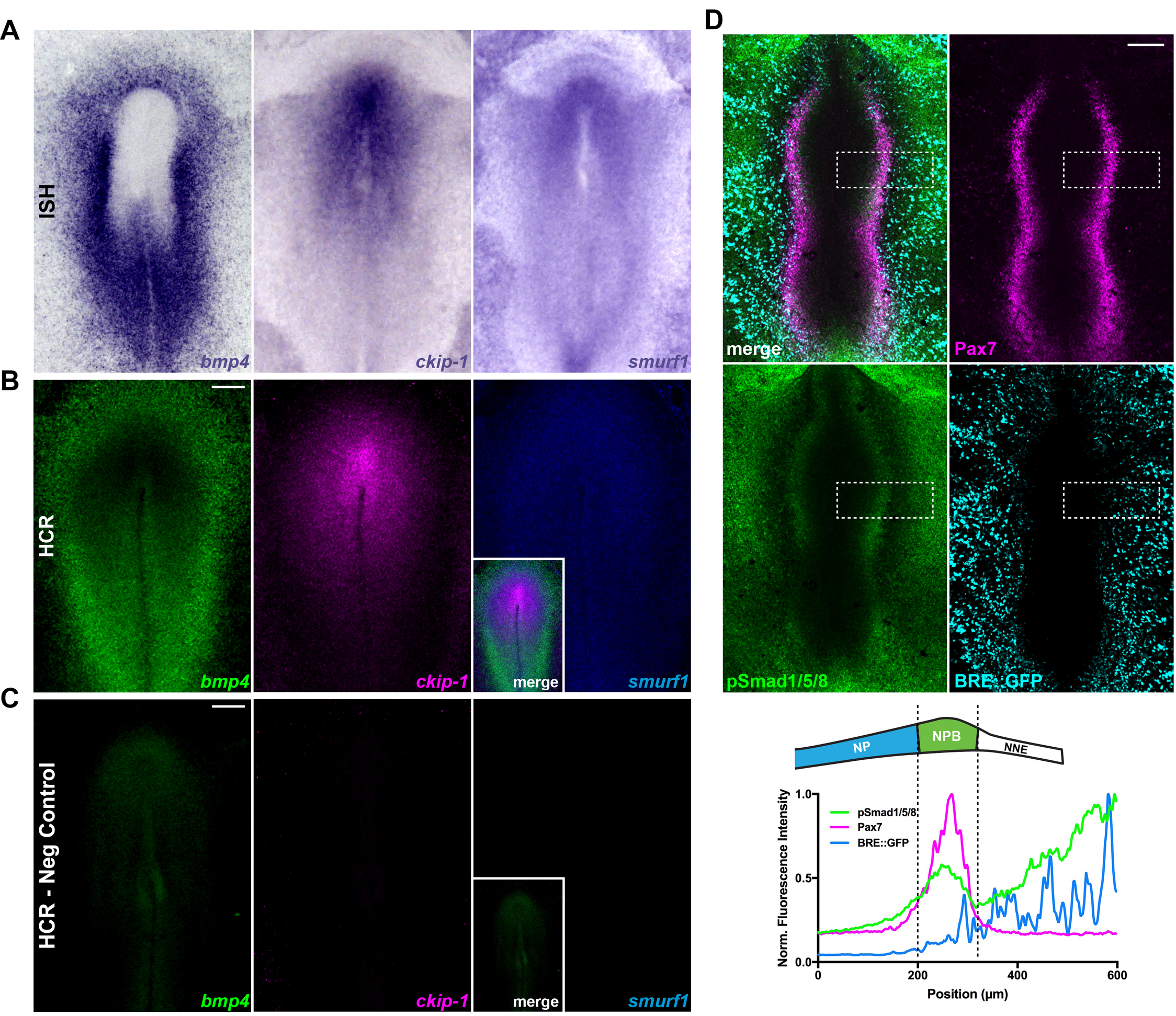

Supplement: S1 Fig — (A) Whole-mount in situ hybridization shows expression of bmp4, ckip-1, and smurf1 in wild-type HH6 chick embryos. (B) HCR showing expression of bmp4, ckip-1, and smurf1 in wild-type HH5 chick embryos. (C) Negative control shows minimal background following HCR protocol. (D) Gastrulating embryos were electroporated with the BRE::GFP reporter (cyan) and then immunostained for pSmad1/5/8 and Pax7. Line traces of staining intensities within the boxed region show intermediate BMP pathway activation in the neural plate border. Underlying data can be found in S1 Data. Scale bars represent 200 μm. BMP, bone morphogenetic protein; BRE::GFP, BMP responsive element–driven green fluorescent protein; HCR, hybridization chain reaction; HH, Hamburger-Hamilton stage; NNE, non-neural ectoderm; NP, neural plate; NPB, neural plate border; Pax7, paired box 7; pSmad1/5/8, phospho-Smads 1/5/8. (TIF) [file pbio.2004425.s001.tif]

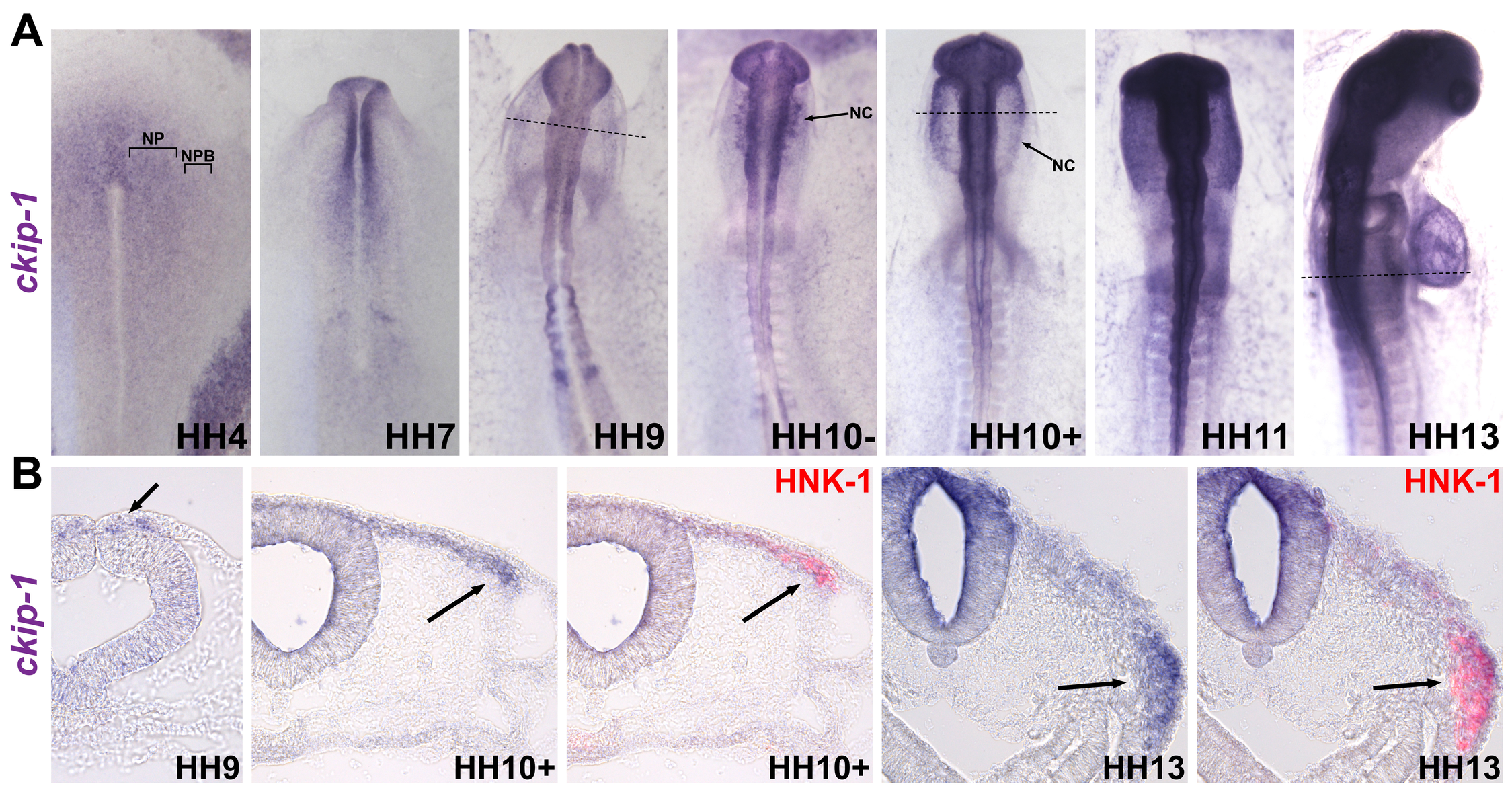

Supplement: S2 Fig — In situ hybridization was performed on wild-type chicken embryos at the indicated HH and are presented in whole-mount dorsal views (A) and transverse sections (B). HNK-1 immunostaining labels migrating NCs in sections. Arrows indicate premigratory NC (HH9) and migratory NC (HH10 and HH13). HH, Hamburger-Hamilton stage; HNK-1, human natural killer 1; NC, neural crest; NP, neural plate; NPB, neural plate border. (TIF) [file pbio.2004425.s002.tif]

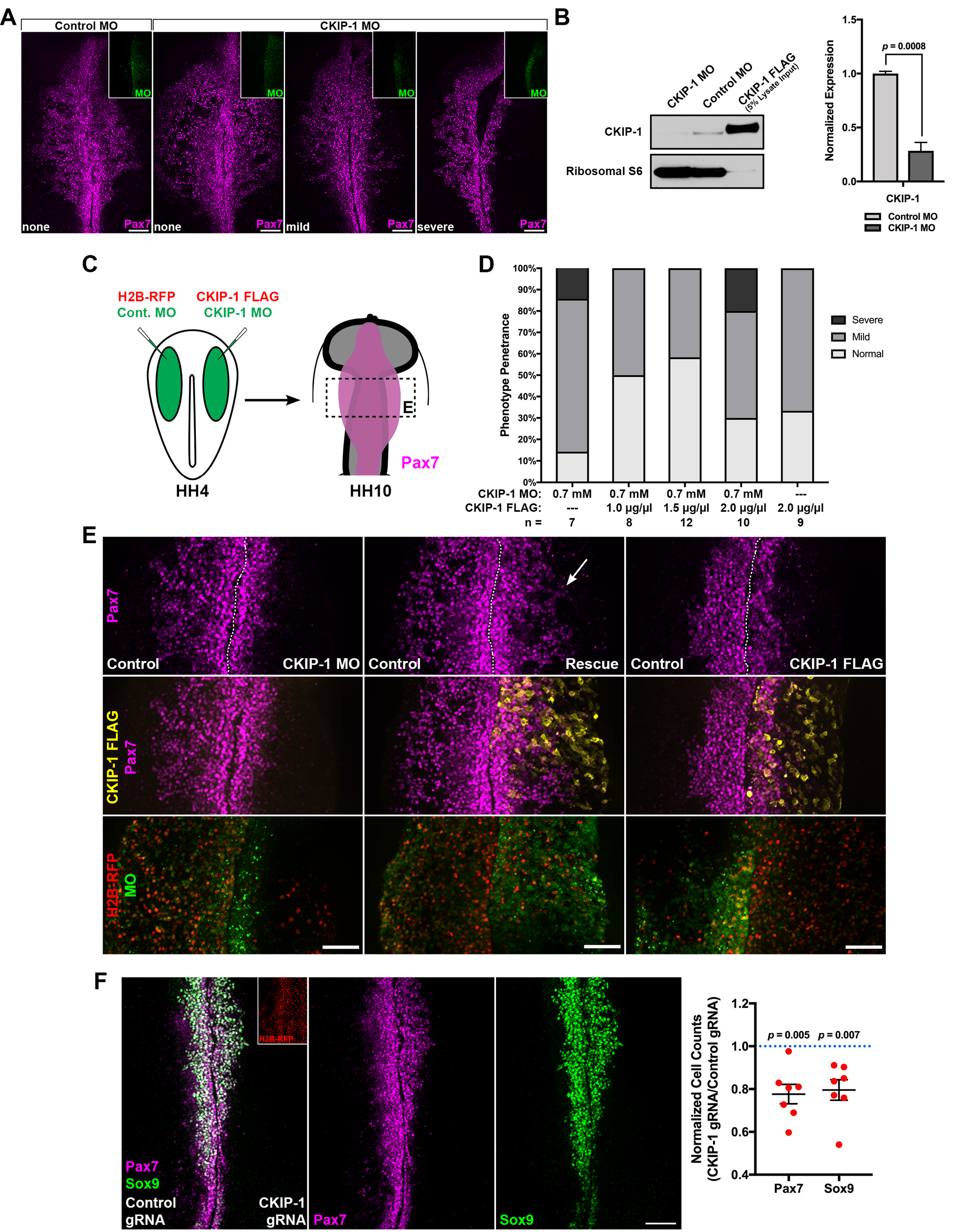

Supplement: S3 Fig — (A) Control MO and CKIP-1 MO electroporated embryos shown at HH10 display ranges of neural crest loss indicated in each panel, as determined by Pax7 immunostaining. Insets show corresponding FITC-conjugated MO electroporation. (B) Western blotting analysis of embryos electroporated with control MO, CKIP-1 MO, or CKIP-1 FLAG and blotted for CKIP-1 and ribosomal S6. The CKIP-1 FLAG lane was loaded with 0.5 μg whole-embryo lysate, while 10 μg was loaded for the other lanes. CKIP-1 intensity was normalized to ribosomal S6 as a loading control; displayed are means ± SEMs. (C) Bilateral electroporations were performed at HH4 with a control mixture (left; control MO and pCI-H2B-RFP) and an experimental mixture (right; doses indicated in D). Resulting embryos were cultured to HH10 and analyzed for Pax7 expression. (D) Quantitation of Pax7 reduction from rescue experiments. (E) Dorsal views of CKIP-1 MO alone (MO at 0.7 mM, H2B-RFP at 2 μg/μl), rescue (CKIP-1 MO at 0.7 mM, CKIP-1 FLAG at 1.5 μg/μl), and CKIP-1 FLAG alone (Control MO at 0.7 mM, CKIP-1 FLAG at 2 μg/μl) embryos displaying Pax7, CKIP-1 FLAG, FITC, and H2B-RFP. Displayed are cropped images as outlined in C. White arrow indicates rescued neural crest cell formation. (F) Embryos were coelectroporated at HH3 with Cas9 protein complexed with nonbinding control or CKIP-1-targeting gRNAs and H2B-RFP as a lineage label. Resulting embryos were incubated to HH9 and processed for Pax7 and Sox9 immunostaining. Cell counts quantitated from whole-mount images demonstrate that CRISPR/Cas9-mediated CKIP-1 knockout phenocopies neural crest loss observed with CKIP-1 MO. Displayed are cell counts normalized to the control side with mean ± SEM. P values from two-tailed Student t test. Scale bars represent 100 μm. Underlying data can be found in S1 Data. Cas9, CRISPR-associated protein 9; CKIP-1, casein kinase interacting protein 1; CRISPR, clustered regularly interspaced short palindromic repeat; FITC, fluorescein isothiocyanate; g [file pbio.2004425.s003.tif]

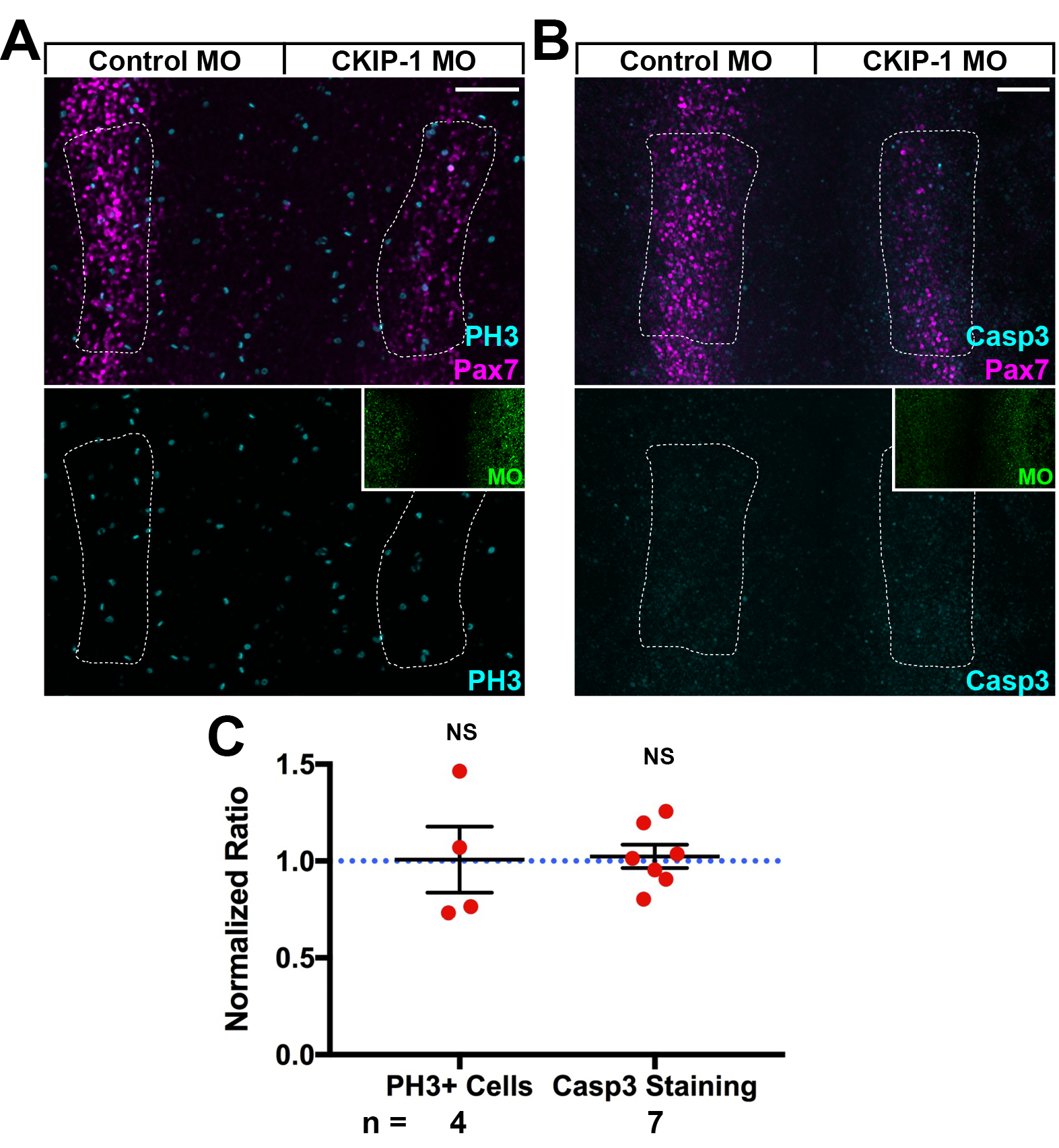

Supplement: S4 Fig — (A,B) CKIP-1 MO–electroporated embryos were harvested at HH7 and then immunostained for Pax7, FITC, and phospho-histone H3 (A) or cleaved-caspase 3 (B). (C) Regions of interest were drawn around the neural plate border (dashed white lines), and phospho-histone H3 cell counts or cleaved-caspase 3 staining intensity was measured and normalized to control and displayed with means ± SEMs. Underlying data can be found in S1 Data. P values from two-tailed Student t test. Scale bars represent 100 μm. CKIP-1, casein kinase interacting protein 1; FITC, fluorescein isothiocyanate; HH, Hamburger-Hamilton stage; MO, morpholino oligonucleotide; NS, not significant; Pax7, paired box 7. (TIF) [file pbio.2004425.s004.tif]

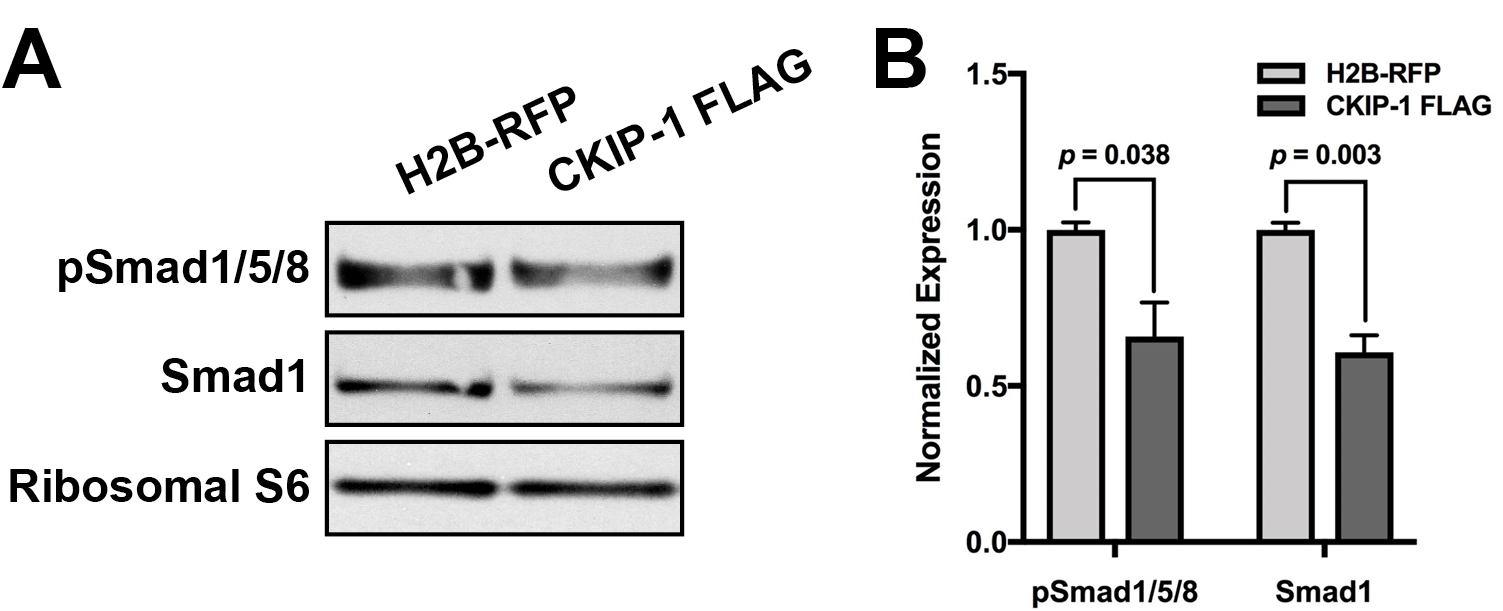

Supplement: S5 Fig — (A) Gastrulating embryos were electroporated with H2B-RFP or CKIP-1 FLAG; then, whole-embryo lysates were prepared at HH7. Ten μg total lysate was loaded per lane, and the resulting blots were processed for the indicated targets, with ribosomal S6 as a loading control. (B) Quantitation of protein expression normalized to ribosomal S6 levels showing means ± SEMs. Underlying data can be found in S1 Data. P values from two-tailed Student t test. CKIP-1, casein kinase interacting protein 1; H2B, histone 2B; HH, Hamburger-Hamilton stage; pSmad1/5/8, phospho-Smads 1/5/8; RFP, red fluorescent protein. (TIF) [file pbio.2004425.s005.tif]
